# The Probiotic Compound VSL#3 Modulates Mucosal, Peripheral, and Systemic Immunity Following Murine Broad-Spectrum Antibiotic Treatment

**DOI:** 10.3389/fcimb.2017.00167

**Published:** 2017-05-05

**Authors:** Ira Ekmekciu, Eliane von Klitzing, Ulrike Fiebiger, Christian Neumann, Petra Bacher, Alexander Scheffold, Stefan Bereswill, Markus M. Heimesaat

**Affiliations:** ^1^Gastrointestinal Microbiology Research Group, Department of Microbiology and Hygiene, Institute for Microbiology and Hygiene, Charité – University MedicineBerlin, Germany; ^2^Department of Cellular Immunology, Clinic for Rheumatology and Clinical Immunology, Charité – University MedicineBerlin, Germany; ^3^German Rheumatism Research Center, Leibniz AssociationBerlin, Germany

**Keywords:** probiotics, antibiotics, innate and adaptive immunity, microbiota, fecal microbiota transplantation, secondary abiotic (gnotobiotic) mice, mucosal and peripheral and central immunity

## Abstract

There is compelling evidence linking the commensal intestinal microbiota with host health and, in turn, antibiotic induced perturbations of microbiota composition with distinct pathologies. Despite the attractiveness of probiotic therapy as a tool to beneficially alter the intestinal microbiota, its immunological effects are still incompletely understood. The aim of the present study was to assess the efficacy of the probiotic formulation VSL#3 consisting of eight distinct bacterial species (including *Streptococcus thermophilus, Bifidobacterium breve, B. longum, B. infantis, Lactobacillus acidophilus, L. plantarum, L. paracasei, and L. delbrueckii* subsp. *Bulgaricus*) in reversing immunological effects of microbiota depletion as compared to reassociation with a complex murine microbiota. To address this, conventional mice were subjected to broad-spectrum antibiotic therapy for 8 weeks and perorally reassociated with either VSL#3 bacteria or a complex murine microbiota. VSL#3 recolonization resulted in restored CD4+ and CD8+ cell numbers in the small and large intestinal lamina propria as well as in B220+ cell numbers in the former, whereas probiotic intervention was not sufficient to reverse the antibiotic induced changes of respective cell populations in the spleen. However, VSL#3 application was as efficient as complex microbiota reassociation to attenuate the frequencies of regulatory T cells, activated dendritic cells and memory/effector T cells in the small intestine, colon, mesenteric lymph nodes, and spleen. Whereas broad-spectrum antibiotic treatment resulted in decreased production of cytokines such as IFN-γ, IL-17, IL-22, and IL-10 by CD4+ cells in respective immunological compartments, VSL#3 recolonization was sufficient to completely recover the expression of the anti-inflammatory cytokine IL-10 without affecting pro-inflammatory mediators. In summary, the probiotic compound VSL#3 has an extensive impact on mucosal, peripheral, and systemic innate as well as adaptive immunity, exerting beneficial anti-inflammatory effects in intestinal as well as systemic compartments. Hence, VSL#3 might be considered a therapeutic immunomodulatory tool following antibiotic therapy.

## Introduction

In the past decades the commensal gut microbiota has been established as an indispensable major key factor in host physiology. The microbiota has been shown to be involved in numerous physiological processes, including vitamin synthesis (LeBlanc et al., 2013), food digestion (Hooper et al., 2002), fat metabolism (Backhed et al., 2004), intestinal angiogenesis (Stappenbeck et al., 2002), enteric nerve function (Husebye et al., 1994), protection from pathogens (Sekirov et al., 2008; Bereswill et al., 2011), and immune system development (Cebra, 1999). Moreover, perturbations of the complex host resident intestinal ecologic system, termed dysbiosis, have been linked to a wide range of pathological conditions including intestinal disorders such as inflammatory bowel diseases (IBD; Baumgart and Carding, 2007), irritable bowel syndrome (IBS; Carroll et al., 2010), and coeliac disease (De Palma et al., 2010), as well as extra-intestinal pathologies such as allergy and asthma (Noverr and Huffnagle, 2004), arthritis (Taurog et al., 1994), type 1 diabetes mellitus (Wen et al., 2008), obesity (Backhed et al., 2007), multiple sclerosis (Ochoa-Reparaz et al., 2009), and distinct cardiovascular diseases (Serino et al., 2014).

With this growing body of evidence concerning the pivotal role of the microbiota in health and disease, the potential of altering and modulating the microbiota composition in beneficial ways has become an increasing focus of attention. Microbiota-modulating intervention strategies include administration of probiotics, defined as live microorganisms conferring a health benefit on the host when administered in adequate amounts (FAO/WHO, 2002). So far, the most emphasis has been laid on investigating the role of probiotic compounds in intestinal inflammation. For instance, *Escherichia coli* strain Nissle 1917 was shown to prevent acute and chronic colitis (Kamada et al., 2005) and to enhance mucosal barrier functions in mice (Ukena et al., 2007; Wassenaar, 2016). Moreover, treating IL-10 deficient mice with *Lactobacillus plantarum* attenuated the severity of colonic inflammation by reducing mucosal IL-12p40 and IFN-γ levels (Schultz et al., 2002). Similarly, the application of VSL#3, a probiotic mixture of eight different bacterial strains (namely *Streptococcus thermophilus, Bifidobacterium breve, B. longum, B. infantis, Lactobacillus acidophilus, L. plantarum, L. paracasei*, and *L. delbrueckii* subsp. *Bulgaricus*) was demonstrated as an effective therapy in both murine IL-10^−/−^ colitis (Jijon et al., 2004) and trinitrobenzenesulphonic acid (TNBS) induced colitis through enhancement of IL-10 and TGF-β expressing T cells (Di Giacinto et al., 2005). Additionally, the induction of TGF-β following oral VSL#3 administration was shown to be effective in ameliorating inflammation in a murine model of T-helper (Th-) 2 cells mediated food allergy (Barletta et al., 2013). The efficacy of probiotic compounds has also been examined in clinical trials with IBD patients. For instance, *E. coli* Nissle 1917 exerted similar efficacy as compared to the established standard medication (i.e., mesalazine) in maintenance therapy of ulcerative colitis (UC) (Kruis et al., 2004). Moreover, a meta-analysis including three controlled trials demonstrated the capability of VSL#3 to induce remission in UC patients (Jonkers et al., 2012). Several studies have also confirmed the protective role of VSL#3 in preventing relapses of pouchitis (Gionchetti et al., 2003), a condition developed by ~50% of UC patients following ileo-anal pouch anastomosis (Shen and Lashner, 2008). In contrast, studies in patients suffering from Crohn's disease (CD) did not unravel a beneficial role of probiotics, neither in induction nor maintenance of remission of this inflammatory disease (Shen et al., 2014).

Several mechanisms to explain the beneficial role of probiotics have been proposed including enhancement of intestinal barrier functions (Ukena et al., 2007), amendment of microbiota diversity, and modulation of the innate and adaptive immune system (Grabig et al., 2006). However, these mechanisms remain in need of further investigation.

In the present study, we focussed on the impact of the commercial probiotic compound VSL#3 on restoring distinct immune cell functions that were affected in mice upon broad-spectrum antibiotic treatment. We therefore performed a comprehensive analysis of the mucosal (i.e., ileal and colonic lamina propria lymphocytes, LPL), peripheral (i.e., mesenteric lymph nodes, MLN) and systemic (i.e., splenic lymphocytes) immune responses in conventional mice with a depleted microbiota following 8 weeks of broad-spectrum antibiotic treatment and upon reassociation with either VSL#3 or fecal microbiota transplantation (FMT) as compared to mice without antibiotic challenge.

## Materials and methods

### Ethics statement

All animal experiments were conducted according to the European Guideline for animal welfare (2010/63/EU) with approval from the commission for animal experiments headed by the “Landesamt für Gesundheit und Soziales” (LaGeSo, Berlin, Germany, registration number G0184/12 and G0097/12).

### Mice

Animals were bred and maintained in the facilities of the “Forschungseinrichtungen für Experimentelle Medizin” (FEM, Charité – Universitätsmedizin, Berlin, Germany) under specific pathogen-free (SPF) conditions. Female age matched C57BL/6j wildtype mice were used.

### Generation of microbiota depleted mice and bacterial recolonization

To eradicate the murine intestinal microbiota 8–10 week old mice were transferred to sterile cages and treated with a quintuple broad-spectrum antibiotic cocktail as previously described (Heimesaat et al., 2006). Three days prior recolonization experiments the antibiotic cocktail was withdrawn and replaced by autoclaved drinking water. For FMT, fresh murine fecal samples were collected from 10 individual female 3 months old naive mice (harboring a conventional SPF microbiota), pooled, dissolved in 10 ml sterile phosphate buffered saline (PBS; Gibco, life technologies, Paisley, UK), and bacterial loads were evaluated by cultural and molecular methods before peroral challenge of mice with 0.3 ml of the suspension by gavage. Another group of mice received an oral suspension of VSL#3 bacteria. VSL#3 is a commercially available probiotic mixture (Manufacturer: SIIT S.r.l, Trezzano sul Naviglio, Italy; distributed by Actial Farmaceutica, Funchal, Madeira, Portugal) consisting of the following eight bacterial species: *Streptococcus thermophilus, Bifidobacterium breve, Bifidobacterium longum, Bifidobacterium infantis, Lactobacillus acidophilus, Lactobacillus plantarum, Lactobacillus paracasei*, and *Lactobacillus delbrueckii* subsp. *Bulgaricus*. A total of 4.5 × 10^11^ probiotic bacteria were dissolved in 50 ml PBS. By gavaging 0.3 ml, each mouse received ~10^9^ viable probiotic bacteria as confirmed by cultural analyses of the suspensions. Mice were continuously kept in a sterile environment (autoclaved food and drinking water or sterile antibiotic cocktail) and were handled under strict aseptic conditions to prevent contaminations.

### Clinical score

To survey clinical signs of inflammation, a standardized cumulative clinical score (maximum 12 points), addressing the occurrence of blood in feces (0: no blood; 2: microscopic detection of blood by the Guajac method using Haemoccult, Beckman Coulter/PCD, Krefeld, Germany; 4: overt blood visible), diarrhea (0: formed feces; 2: pasty feces; 4: liquid feces), and the clinical aspect (0: normal; 2: ruffled fur, less locomotion; 4: isolation, severely compromized locomotion, pre-final aspect) was applied daily as described earlier (Haag et al., 2012).

### Sampling procedures

Mice were sacrificed by isofluran treatment (Abbott, Greifswald, Germany) at day (d) 28 post recolonization. Tissue samples from spleen, MLN, ileum and colon, and luminal samples from colon were removed under sterile conditions. Ileal and colonic *ex vivo* biopsies were collected from each mouse in parallel for immunological, microbiological, and immunohistochemical analysis. For immunohistochemical stainings, ileum and colon samples were immediately fixed in 5% formalin and embedded in paraffin, and sections (5 μm) were stained with the respective antibodies as described below.

### Bacterial colonization densities following recolonization of secondary abiotic mice with VSL#3 or complex murine microbiota

Total intestinal loads of VSL#3 bacteria were quantified in serial dilutions of fecal and large intestinal luminal samples streaked onto Columbia-Agar supplemented with 5% sheep blood and Columbia-CNA Agar supplemented with colistin and nalidixic acid (both Oxoid) in parallel and incubated under aerobic (with 5% CO_2_), microaerophilic (in jars using CampGen gas packs; Oxoid) and obligate anaerobic (in jars using Anaerogen gas packs; Oxoid) conditions for at least 2 days. Bacterial species were identified according to their typical morphological appearances. The total VSL#3 bacterial loads of intestinal samples were approximated as the sum of identified colony forming units (CFU) derived from the respective culture conditions. The detection limit of viable bacteria was ≈100 CFU per g.

The complex intestinal microbiota composition in conventionally colonized SPF mice and mice subjected to FMT was assessed by quantitative 16S rRNA based real time PCR as described previously (Heimesaat et al., 2010, 2014; Rausch et al., 2013; Thoene-Reineke et al., 2014).

### Immunohistochemistry

*In situ* immunohistochemical analysis of ileal and colonic paraffin sections was performed as previously described (Heimesaat et al., 2007, 2010; Alutis et al., 2015). Primary antibodies against cleaved caspase-3 (Asp175, Cell Signaling, Beverly, MA, USA, 1:200), Ki67 (TEC3, Dako, Glostrup, Denmark, 1:100), CD3 (#N1580, Dako, 1:10), FOXP3 (FJK-16s, eBioscience, San Diego, CA, USA, 1:100), and B220 (eBioscience, 1:200) were used. For detection, the LSAB method was applied with FastRed (Dako) as chromogen. For each animal, the average number of positively stained cells within at least six high power fields (HPF, 400× magnification) was determined microscopically by a blinded investigator.

### Lymphocytes isolation from spleens and mesenteric lymph nodes

Single cell suspensions were generated from spleens and MLN, and erythrocytes were removed from splenic samples by 1.66% ammonium chloride. All samples were resuspended in defined volumes of PBS/0.5% BSA and subjected to further processing (Cording et al., 2013).

### LPL isolation

Segments of the murine gut were removed and freed from fat, connective tissue and PP, cut longitudinally and cleared from luminal content and mucus with ice-cold PBS. The isolation of LPL followed a standard protocol with minor modifications (Sheridan and Lefrancois, 2012). Briefly, the intestines were cut into 0.5 cm pieces and incubated twice with 25 ml Hanks balanced salt solution (HBSS; Gibco) containing 1 mM dithioerythritol (DTE; Carl Roth) for 20 min at 37°C and 220 rpm. Afterwards the intestines were introduced to HBSS containing 1.3 mM ethylenediaminetetraacetic acid (EDTA; Life Technologies, Eugene, Oregon, USA). Subsequently the cells were placed in digestion solution, containing 0.5 mg/ml collagenase A (Roche, Mannheim, Germany), 0.5 mg/ml DNAse I (Roche), 10% FCS, and 1 mM of each CaCl2 and MgCl_2_ (both Carl Roth). Digestion was performed through incubation for 45 min at 37° and 220 rpm. After the incubation the digested tissues were washed with RPMI containing 5% FCS, resuspended in 5 ml 44% Percoll (GE Healthcare, Uppsala, Sweden), and overlaid on 5 ml 67% Percoll in a 15 ml Falcon tube. Percoll gradient separation was performed by centrifugation at 600 g for 20 min at room temperature. LPL were collected from the interphase, washed once and suspended in PBS/0.5% BSA.

### Surface and intracellular stainings and flow cytometry

Surface staining was performed using the following antibodies: FITC-anti-CD4 (Clone RM4-5; 1:200), PerCP-anti-CD8 (Clone 53-6.7; 1:100), PacBlue-anti-B220 (Clone RA3-6B2, 1:200), APC-Cy7-anti-CD25 (Clone PC61, 1:200), PE-anti-CD44 (Clone IM7, 1:200), APC-anti-CD86 (Clone B7-2, 1:200; all from BD Biosciences, San Jose, CA, USA).

For intracellular staining cells from spleen, MLN and intestinal LP were restimulated for 5 h with 10 ng/ml phorbol myristate acetate (PMA) and 1 μg/ml ionomycin, in a tissue culture incubator at 37°C (both Sigma-Aldrich). Ten micrograms per microliter of brefeldin A (Sigma-Aldrich) was added to the cell suspensions after 1 h of polyclonal restimulation. Then cells were treated with LIVE/DEAD Fixable Aqua Dead Cell Stain kit (life technologies) and hereafter fixed with 2% paraformaldehyde (PFA; Sigma-Aldrich) for 20 min at room temperature. Cells were stained in 0.5% saponin (Sigma-Aldrich) using the following antibodies: PacBlue-Anti-CD4 (Clone RM4-5; 1:400), PE-Cy7-anti-IFN-γ (Clone XMG 1.2; 1:400), (both from BD Biosciences) FITC-anti-IL17A (Clone TC11-18H10.1; 1:200, BioLegend, San Diego, CA, USA), PE-anti-IL10 (Clone JESS-16E3; 1:100), APC-anti-IL22 (Clone IL22JOP; 1:100) (both from eBioscience). All data were acquired on a MACSQuant analyzer (Miltenyi Biotec, Bergisch Gladbach, Germany) and analyzed with FlowJo Software v10.1 (Tree star, Ashland, OR, USA).

### Real-time PCR

RNA was isolated from snap frozen ileal and colonic *ex vivo* biopsies, reverse transcribed, and analyzed for cytokine specific mRNA as described earlier (Munoz et al., 2009). Murine IL-22, IL-17A, IL-10, and IFN-γ mRNA expressions were analyzed using Light Cycler Data Analysis Software (Roche). Expression levels were calculated relative to the housekeeping gene for hypoxanthine-phosphoribosyltransferase expression and indicated as “Arbitrary Units” (fold expression).

### Statistical analysis

Medians and significance levels using appropriate tests as indicated (Mann Whitney *U*-test and one-way ANOVA with Tukey's *post-hoc* test for multiple comparisons) were determined using GraphPad Prism Software v6 (La Jolla, CA, USA). Two-sided probability (*p*) values ≤ 0.05 were considered significant. All experiments were repeated at least twice.

## Results

### Macroscopic and microscopic intestinal changes in secondary abiotic mice upon recolonization with VSL#3 or fecal microbiota transplantation

**Figure 1:**
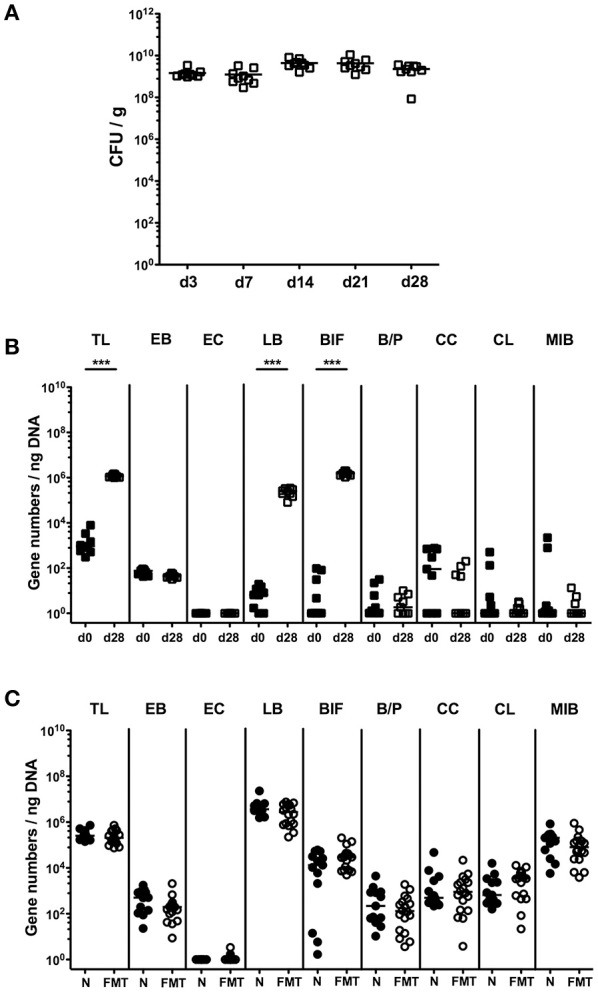
**Bacterial colonization densities following bacterial recolonization of secondary abiotic mice with VSL#3 or complex murine microbiota**. Secondary abiotic mice were generated by broad-spectrum antibiotic treatment and perorally reassociated with the probiotic compound VSL#3 or subjected to fecal microbiota transplantation. **(A)** Bacterial colonization densities were assessed in fecal samples (CFU/g, colony forming units per gram) over time upon reassociation by culture on day (d) 0, 3, 7, 14, 21, 28 following peroral challenge with the probiotic compound VSL#3 (white squares). **(B)** Changes in intestinal microbiota composition of secondary abiotic mice (d0, closed squares) within 28 days following peroral VSL#3 reassociation (d28, open squares) or **(C)** fecal microbiota transplantation (FMT, white circles) were assessed in fecal samples by quantitative Real-Time PCR amplifying bacterial 16S rRNA variable regions of the main intestinal bacterial groups and expressed as 16S rRNA gene numbers per ng DNA: TL, Total eubacterial load; EB, *Enterobacteriaceae*; EC, *Enterococcus* spp.; LB, Lactic acid bacteria; BIF, Bifidobacteria; BP, *Bacteroides/Prevotella* spp.; CC, *Clostridium coccoides* group; CL, *Clostridium leptum* group; and MIB, *Mouse Intestinal Bacteroides*. Conventional, naive (N, black circles) mice served as controls. Medians (black bars) and significance levels determined by Mann Whitney *U*-test (^***^p < 0.001) are indicated. Data were pooled from two independent experiments.

Given a better acceptance by the patients and more practical mode of peroral application of a probiotic formulation as compared to FMT in both ambulant and hospital settings, we compared the immunopathological impact of the probiotic compound VSL#3 and complex microbiota in the with broad-spectrum antibiotics treated host. Therefore, we virtually depleted the intestinal microbiota of conventional mice by quintuple antibiotic treatment. These secondary abiotic (ABx) mice then received either 10^9^ viable VSL#3 bacteria via the oral route or were subjected to FMT by gavage, whereas naive, conventionally colonized and ABx mice served as positive and negative controls, respectively. From day 3 until day 28 following VSL#3 peroral challenge, mice could be stably colonized as indicated by 10^9^ CFU of VSL#3 bacteria per g feces (Figure 1A). In order to exclude that upon cessation of antibiotic treatment and peroral reconstitution with VSL#3 remnant commensal bacteria might grow back, we surveyed the intestinal microbiota composition applying highly sensitive and culture-independent 16S rRNA based molecular quantitative RT-PCR. In fact, only bifidobacteria and lactobacilli as main bacterial constituents of the applied probiotic compound increased in fecal samples until day 28 following probiotic challenge (*p* < 0.001; Figure 1B), whereas the other intestinal bacterial commensals remained virtually unchanged (n.s.; Figure 1B). Moreover, mice subjected to FMT showed at day 28 after recolonization a complex large intestinal microbiota composition that was comparable to the microbiota in conventional control animals (Figure 1C).

**Figure 2:**
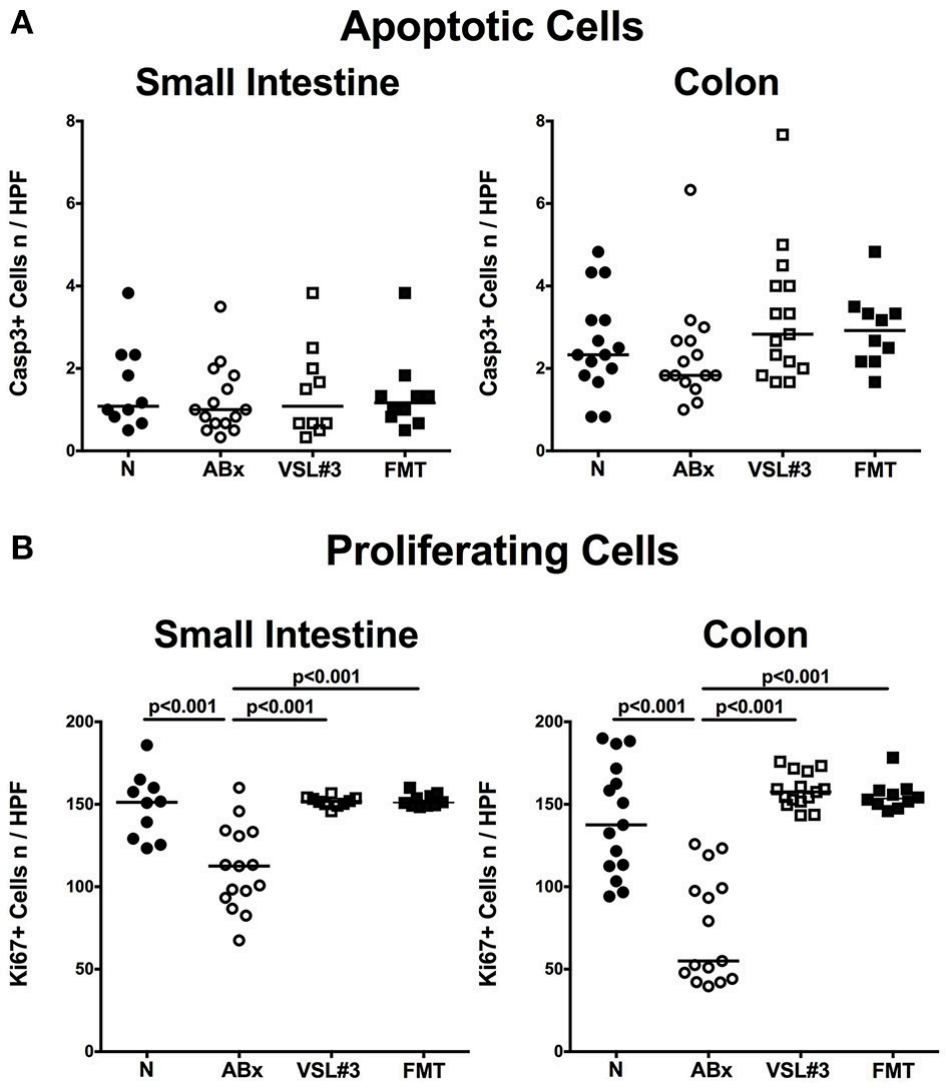
**Small intestinal and colonic epithelial apoptotic and proliferating cells following recolonization of secondary abiotic mice with VSL#3 or complex murine microbiota**. The average numbers of **(A)** apoptotic (positive for Casp3) and **(B)** proliferating cells (positive for Ki67) in at least six representative high power fields (HPF, 400× magnification) per animal were determined in immunohistochemically stained small intestinal and colonic tissue of naive conventional mice (N), through antibiotic treatment generated secondary abiotic mice (ABx), and mice subjected to VSL#3 or fecal microbiota transplantation (FMT) on day 28 following peroral reassociation. Medians (black bars) and significance levels (*p*-values) determined with one-way ANOVA test followed by Tukey post-correction test for multiple comparisons are indicated. Data shown were pooled from two independent experiments (*n* = 10–15/group).

Given that mice were clinically/macroscopically uncompromised upon antibiotic treatment as well as following respective recolonization regimens as assessed by a clinical scoring system on a daily basis (not shown), we next assessed potential microscopic changes in the intestinal tract. To address this, we quantitatively surveyed apoptotic cell numbers in small and large intestinal epithelia applying *in situ* immunohistochemistry. In line with the uncompromised clinical conditions, apoptosis was neither induced by broad-spectrum antibiotic treatment nor by respective peroral reassociation (n.s.; Figure 2A; Figure S1). Numbers of epithelial cells positive for Ki67, a sensitive marker for cell proliferation and regeneration (Scholzen and Gerdes, 2000), however, were significantly reduced in both, ileum and colon following broad-spectrum antibiotic treatment. Notably, administration of either VSL#3 or complex SPF microbiota was sufficient to restore regenerative epithelial properties as indicated by higher small as well as large intestinal Ki67+ cell numbers as compared to ABx mice (*p* < 0.001), that were comparable to those observed in naive SPF mice (Figure 2B; Figure S1).

### Adaptive immune cell subsets in small and large intestines *in situ* following broad-spectrum antibiotic treatment and recolonization with VSL#3 or fecal microbiota transplantation

**Figure 3:**
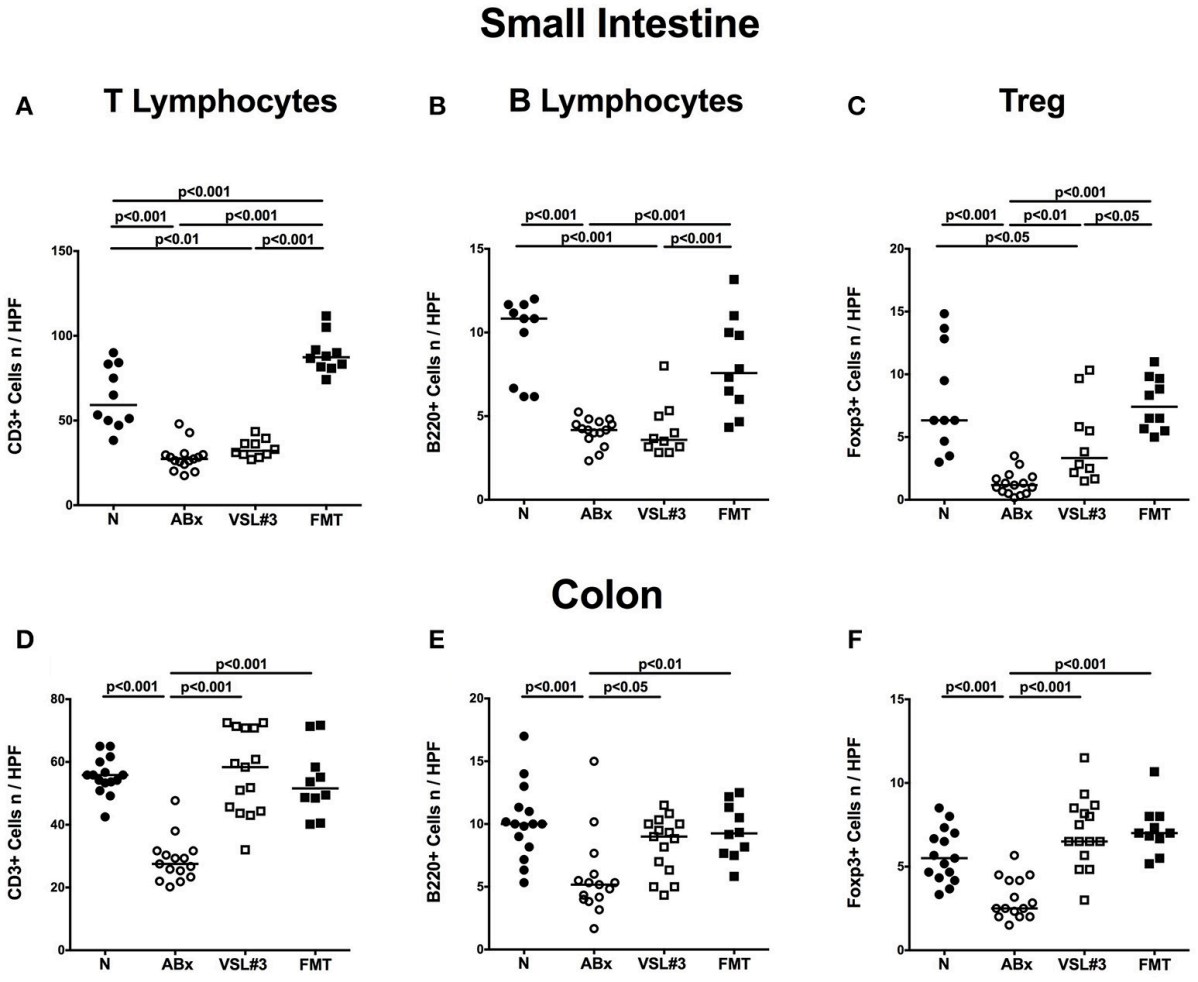
**Adaptive immune cell subsets in small and large intestines *in situ* following recolonization of secondary abiotic mice with VSL#3 or complex murine microbiota**. The average numbers of T lymphocytes (positive for CD3, **A,D**), B lymphocytes (positive for B220, **B,E**), and regulatory T cells (positive for FOXP3, **C,F**) in the small intestinal (upper panel, **A–C**) and colonic (lower panel, **D–F**) tissue in at least six representative high power fields (HPF, 400× magnification) per animal were determined in immunohistochemically stained tissues of naive conventional mice (N), by antibiotic treatment generated secondary abiotic mice (ABx), and mice subjected to VSL#3 recolonization or fecal microbiota transplantation (FMT) on day 28 following peroral reassociation. Medians and significance levels (*p*-values) determined with one-way ANOVA test followed by Tukey post-correction test for multiple comparisons are indicated. Data shown were pooled from two independent experiments (*n* = 10–15/group).

To further dissect the role of VSL#3 in modulating adaptive immune responses following microbial depletion, we quantified cell numbers of distinct immune cell populations in both small and large intestines of mice at day 28 post recolonization with either VSL#3 or FMT by *in situ* immunohistochemical staining of paraffin sections (Figure 3; Figure S1). Broad-spectrum antibiotic treatment was associated with reduced numbers of CD3+ T lymphocytes (*p* < 0.001; Figures 3A,D; Figure S1), B220+ B lymphocytes (*p* < 0.001; Figures 3B,E; Figure S1), and Foxp3+ Treg (*p* < 0.001; Figures 3C,F; Figure S1) in mucosa and lamina propria of both ileum and colon. Application of either VSL#3 or complex SPF microbiota, however, was sufficient to restore adaptive immune cell populations in the colon as indicated by T and B cell as well as Treg numbers that were comparable to naive mice at day 28 following respective recolonization (Figures 3D–F; Figure S1). Numbers of T and B lymphocyte numbers increased in small intestines of secondary abiotic mice following FMT, but not VSL#3 recolonization (*p* < 0.001; Figures 3A,B; Figure S1). Small intestinal Treg numbers were elevated upon either reassociation regimen and to the highest extent by FMT (Figure 3C; Figure S1).

To exclude that the observed effects were microbiota driven and not due to antibiotic withdrawal *per se*, we quantitatively assessed respective intestinal immune cell populations in secondary abiotic mice 4 weeks following cessation of broad-spectrum antibiotic treatment (Figure S2). However, withdrawal of antibiotic treatment (ABx%) did not restore any of the analyzed small or large intestinal immune cell populations as indicated by comparable cell numbers in ABx and ABx% mice (n.s.; Figure S2).

Hence, depending on the intestinal compartment, peroral application of VSL#3 or complex murine microbiota could sufficiently reverse antibiotics-induced decreases in intestinal immune cell populations with the most prominent effect in the colon.

### Distinct T cell populations in intestinal and systemic compartments of secondary abiotic mice following recolonization with VSL#3 or fecal microbiota transplantation

**Figure 4:**
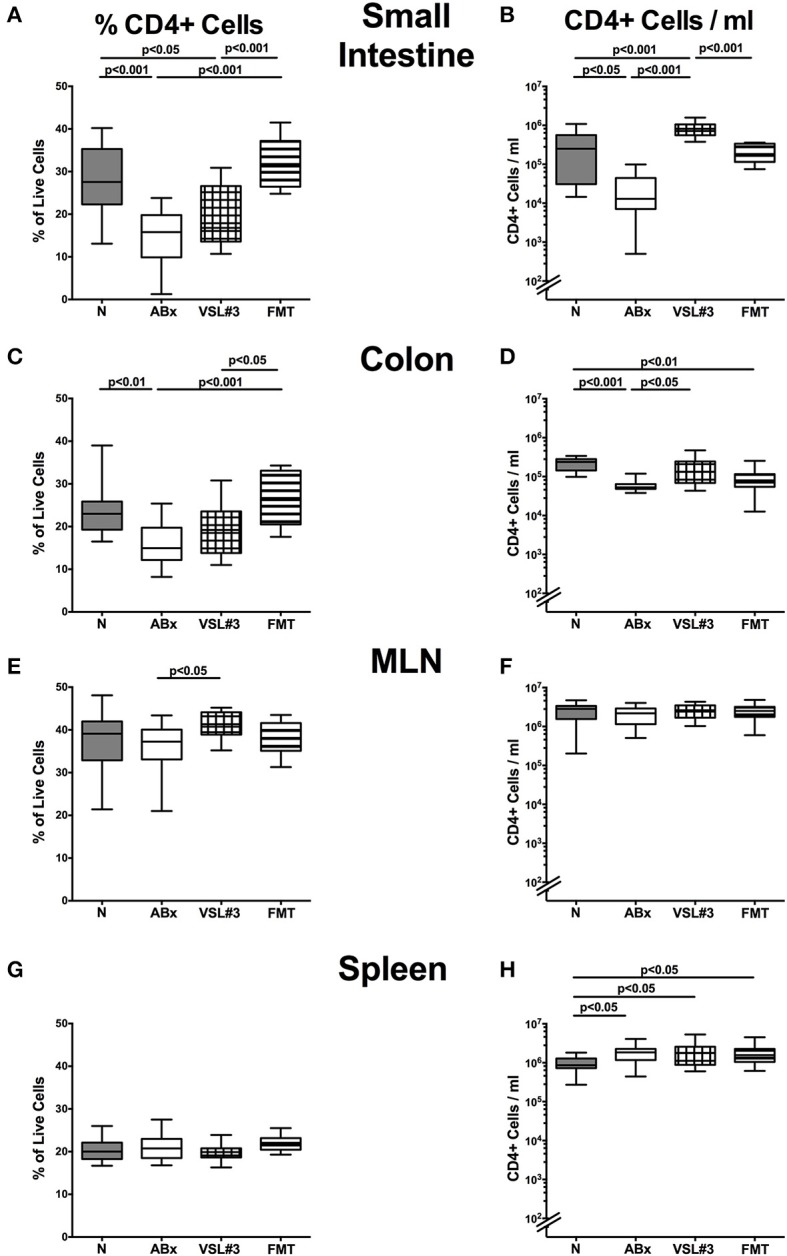
**CD4+ cells in intestinal and systemic compartments of secondary abiotic mice following recolonization with VSL#3 or complex murine microbiota**. Conventionally colonized adult mice were treated with broad-spectrum antibiotics for 8 weeks and differentially recolonized by gavage thereafter. Subsequently, lymphocytes from small intestinal and colonic lamina propria, MLN and spleen were isolated, and analyzed by flow cytometry. The percentages (left panel **A,C,E,G**) and absolute cell numbers (right panel **B,D,F,H**) of the CD4+ lymphocyte population in small intestine **(A,B)**, colon **(C,D)**, MLN **(E,F)**, and spleen **(G,H)** in naive conventional mice (N), by antibiotic treatment generated secondary abiotic mice (ABx), and mice subjected to VSL#3 recolonization or fecal microbiota transplantation (FMT) were determined on day 28 following peroral reassociation. Box plots represent the 75th and 25th percentiles of the medians (black bar inside the boxes). Total range and significance levels (*p*-values) determined with one-way ANOVA test followed by Tukey post-correction test for multiple comparisons are indicated. Data shown were pooled from two independent experiments (*n* = 10–15/group).

**Figure 5:**
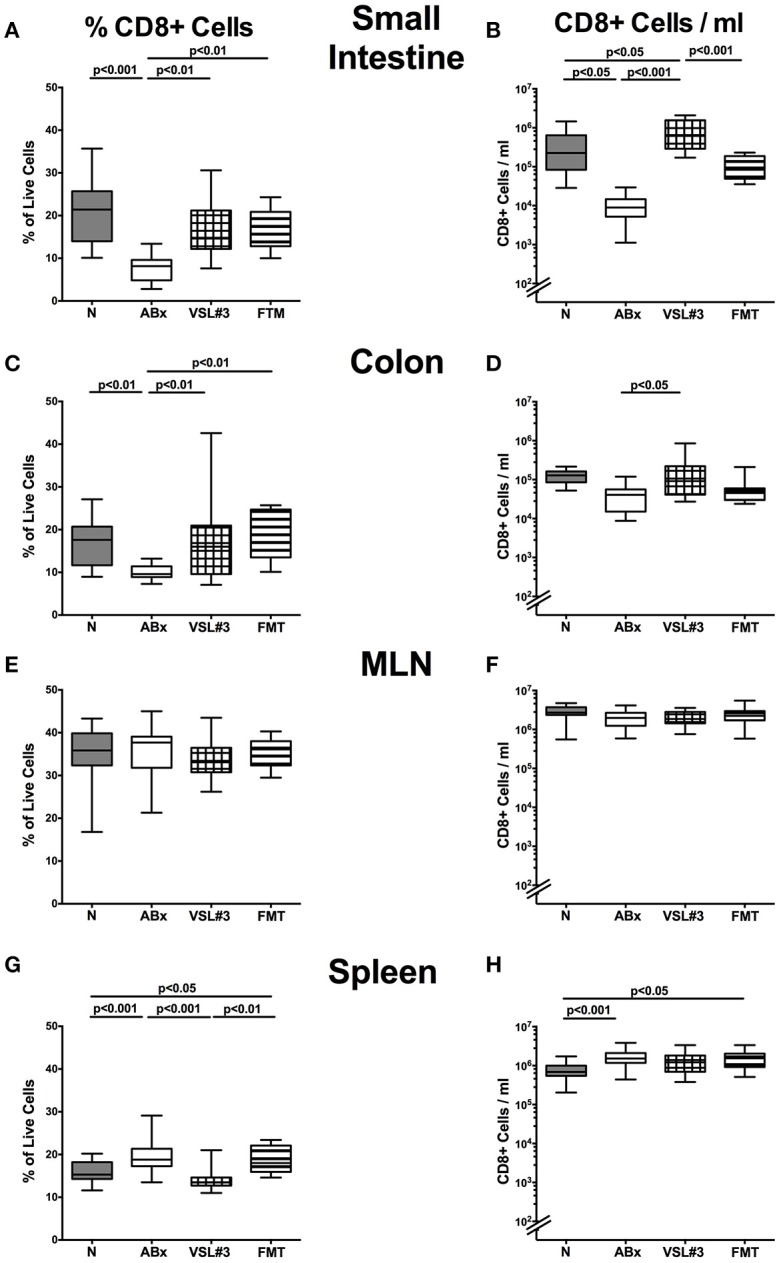
**CD8+ cells in intestinal and systemic compartments of secondary abiotic mice following recolonization with VSL#3 or complex murine microbiota**. The percentages (left panel **A,C,E,G**) and absolute cell numbers (right panel **B,D,F,H**) of the CD8+ lymphocyte population of small intestine **(A,B)**, colon **(C,D)**, MLN **(E,F)**, and spleen **(G,H)** in naive conventional mice (N), by antibiotic treatment generated secondary abiotic mice (ABx), and mice subjected to VSL#3 recolonization or fecal microbiota transplantation (FMT) were determined on day 28 following peroral reassociation. Box plots represent the 75th and 25th percentiles of the medians (black bar inside the boxes). Total range and significance levels (*p*-values) determined with one-way ANOVA test followed by Tukey post-correction test for multiple comparisons are indicated. Data shown were pooled from two independent experiments (*n* = 10–15/group).

**Figure 6:**
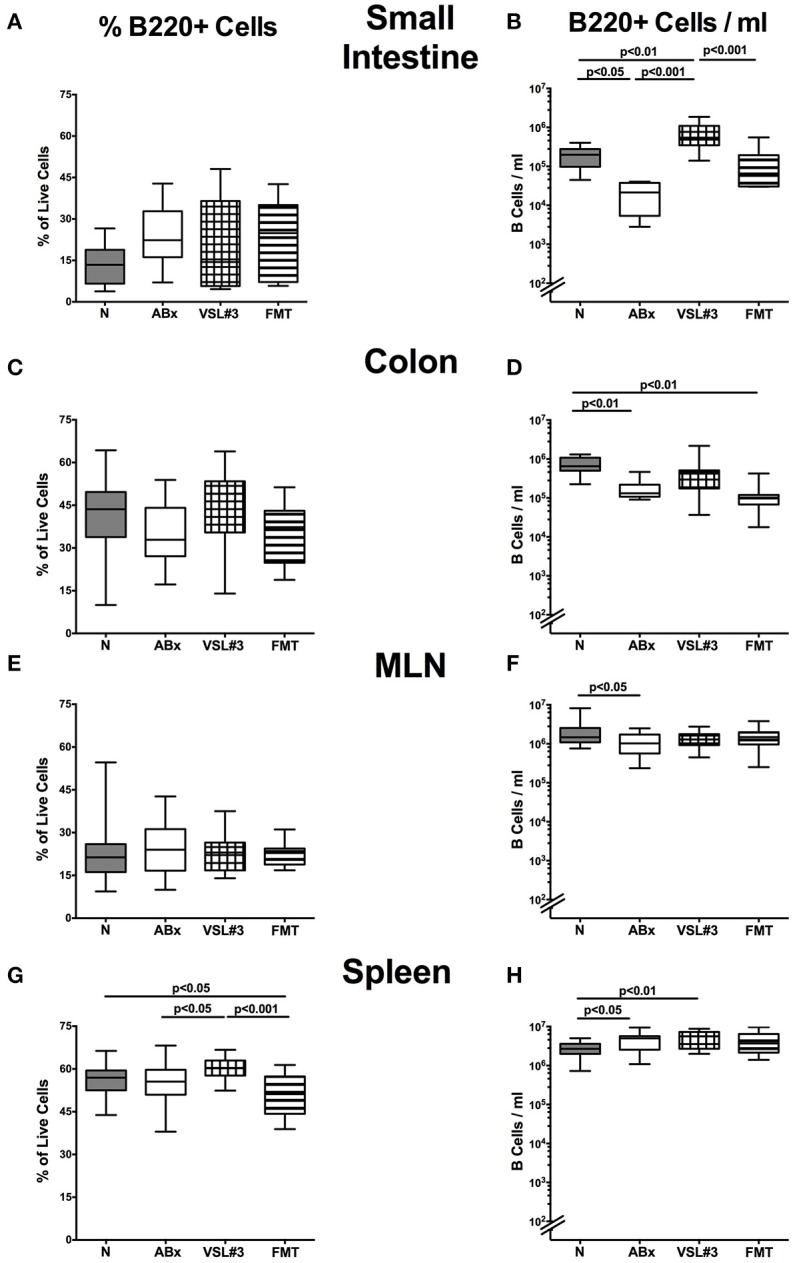
**B220+ cells in intestinal and systemic compartments of secondary abiotic mice following recolonization with VSL#3 or complex murine microbiota**. The percentages (left panel **A,C,E,G**) and absolute cell numbers (right panel **B,D,F,H**) of the B220+ lymphocyte population of small intestine **(A,B)**, colon **(C,D)**, MLN **(E,F)**, and spleen **(G,H)** in naive conventional mice (N), by antibiotic treatment generated secondary abiotic mice (ABx), and mice subjected to VSL#3 recolonization or fecal microbiota transplantation (FMT) were determined on day 28 following peroral reassociation. Box plots represent the 75th and 25th percentiles of the medians (black bar inside the boxes). Total range and significance levels (*p*-values) determined with one-way ANOVA test followed by Tukey post-correction test for multiple comparisons are indicated. Data shown were pooled from two independent experiments (*n* = 10–15/group).

We next elaborated the capacities of peroral VSL#3 application or FMT to induce, maintain and modulate distinct immune cell populations in mucosal, peripheral, and systemic immunological sites of mice that had been treated with broad-spectrum antibiotics. To address this, we performed flow-cytometric analyses of lymphocytes derived from the lamina propria of small and large intestine, MLN and spleen of mice at day 28 post-recolonization. The gating strategies are depicted in Figures S3A–F. We firstly focused on relative abundances and absolute numbers of the main lymphocytic groups, namely CD4+ (Figures 4A–H) and CD8+ (Figures 5A–H) T lymphocytes as well as B220+ B lymphocytes (Figures 6A–H). Antibiotic treatment resulted in decreased relative abundances and absolute numbers of CD4+ T helper cells in both the small and large intestines (*p* < 0.05–0.001; Figures 4A–D), whereas VSL#3 administration could sufficiently restore respective cell numbers at either mucosal site. Furthermore, abundances of CD4+ cells increased in MLN upon VSL#3 treatment (*p* < 0.05; Figure 4E). Following FMT, CD4+ cell frequencies were higher in small and large intestines as compared to ABx mice (*p* < 0.001; Figures 4A,C). Whereas frequencies of splenic CD4+ cells were rather unchanged upon antibiotic treatment and peroral reassociation (n.s.; Figure 4G), increased CD4+ cell numbers could be observed in the spleens of mice that had undergone antibiotic therapy, regardless whether subsequently recolonized or not (*p* < 0.05; Figure 4H).

We further analyzed CD8+ cytotoxic T cell responses in intestinal and systemic compartments upon antibiotic treatment and subsequent bacterial reassociation. Both VSL#3 treatment and FMT could sufficiently restore the antibiotics induced CD8+ cell frequency reduction in the small and large intestines as indicated by higher small and large intestinal CD8+ cell abundances as compared to ABx mice (*p* < 0.01; Figures 5A,C), which also held true for absolute CD8+ cell numbers upon VSL#3 treatment (*p* < 0.05–0.001; Figures 5B,D). Whereas CD8+ cells were rather unchanged in MLN of ABx mice with and without bacterial recolonization, both frequencies and absolute numbers of splenic CD8+ cells increased upon broad-spectrum antibiotic treatment (*p* < 0.001; Figures 5G,H). Interestingly, VSL#3, but not FMT could reverse this effect on CD8+ cell abundances (*p* < 0.001 vs. ABx mice; Figure 5G). Hence, again peroral VSL#3 application or FMT were able to reverse antibiotics induced decreases in T cells, depending on the respective immunological compartment.

### B lymphocytes in intestinal and systemic compartments of secondary abiotic mice following recolonization with VSL#3 or fecal microbiota transplantation

We next expanded our comprehensive survey on lymphocyte populations during antibiotic treatment and bacterial recolonization to B220+ B cells. Whereas decreased B220+ cell counts were detected in small and large intestines as well as in MLN following broad-spectrum antibiotic treatment (*p* < 0.05–0.01; Figures 6B,D,F), VSL#3, but not FMT resulted in elevated small intestinal B lymphocytes (*p* < 0.001 vs. ABx; Figure 6B). In the splenic compartment, B220+ cell numbers increased following antibiotic treatment, but also upon additional VSL#3 challenge (*p* < 0.05 and *p* < 0.01, respectively; Figure 6H). In addition, B220+ cells were more abundant in the spleen of VSL#3 as compared to untreated ABx mice (*p* < 0.05; Figure 6G). Taken together, our data indicate that an intestinal VSL#3 microbiota is capable of inducing and modulating distinctive immune cell populations, thus antagonizing immunological consequences of antibiotic treatment not only at mucosal site, but, to some extent, also on a systemic level of the immune system.

### Regulatory T cells and dendritic cells in intestinal and systemic compartments of secondary abiotic mice following recolonization with VSL#3 or fecal microbiota transplantation

**Figure 7:**
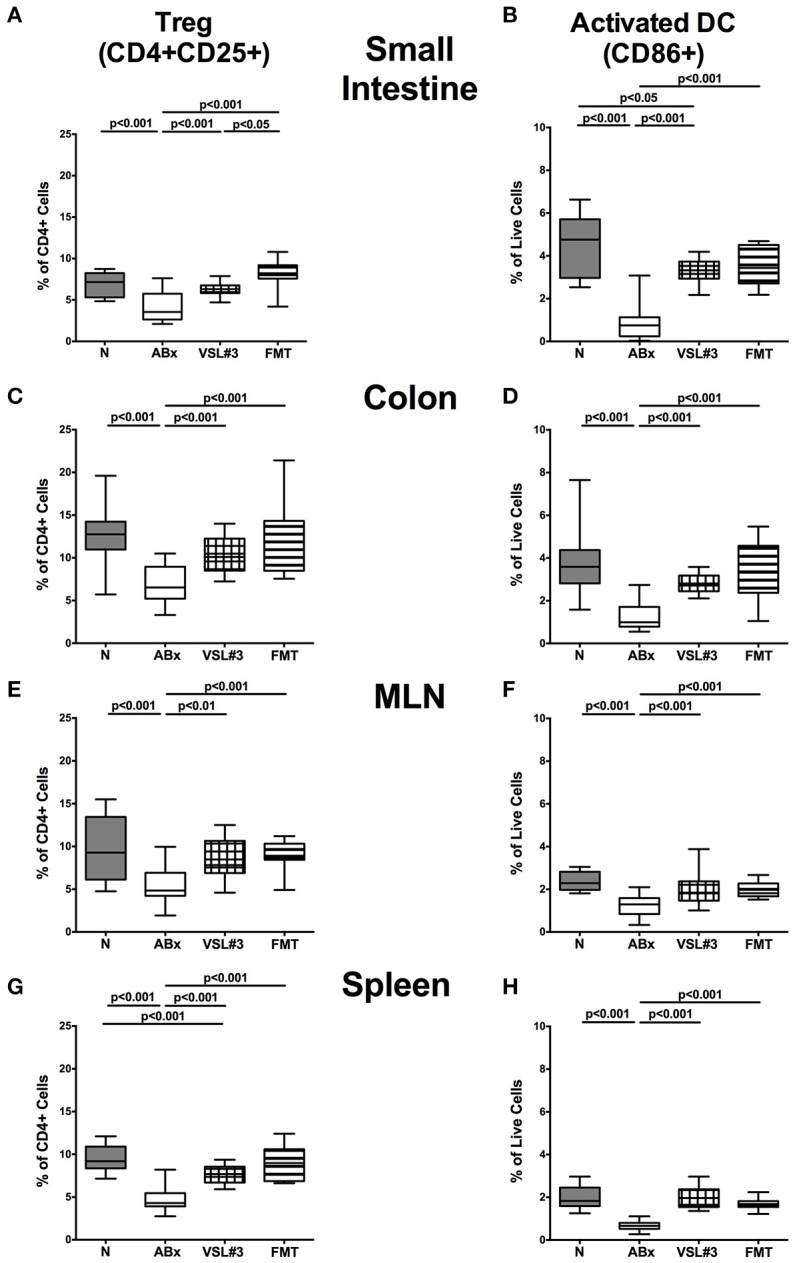
**Regulatory T cells and activated dendritic cells in intestinal and systemic compartments of secondary abiotic mice following recolonization with VSL#3 or complex murine microbiota**. The frequencies of regulatory T cells (Treg, CD4+CD25+, gated on CD4+ cells) (left panel **A,C,E,G**) and activated dendritic cells (CD86+, gated on live CD4-CD8-cells, right panel **B,D,F,H**) in the small intestine **(A,B)**, colon **(C,D)**, MLN **(E,F)**, and spleen **(G,H)** in naive conventional mice (N), by antibiotic treatment generated secondary abiotic mice (ABx), and mice subjected to VSL#3 recolonization or fecal microbiota transplantation (FMT) were determined on day 28 following peroral reassociation. Box plots represent the 75th and 25th percentiles of the medians (black bar inside the boxes). Total range and significance levels (*p*-values) determined with one-way ANOVA test followed by Tukey post-correction test for multiple comparisons are indicated. Data shown were pooled from two independent experiments (*n* = 10–15/group).

In the following, we addressed whether recolonization with VSL#3 or FMT following broad-spectrum antibiotic treatment was associated with changes in defined T cell subsets and in the activation status of distinct cell populations. We therefore stained CD4+ cells with antibodies against CD25, a surface protein characteristic for Treg. Microbial depletion by antibiotic treatment led to reduced abundances of the CD4+CD25+ subpopulation in all intestinal and systemic immunological compartments under investigation (*p* < 0.001; Figure 7). Remarkably, VSL#3 administration as well as FMT enhanced Treg abundances and completely reversed the antibiotics induced effect (*p* < 0.01–0.001 vs. ABx; Figures 7A,C,E,G). VSL#3-induced splenic abundances of CD4+CD25+ cells were, however, less distinct than in naive controls (*p* < 0.001; Figure 7G). Broad-spectrum antibiotic treatment was further accompanied by a down-regulation of the surface marker CD86, a co-stimulatory molecule marking activated DC (Wallet et al., 2005), in small intestine, colon, MLN and spleen of ABx mice, whereas both VSL#3 treatment and FMT virtually reversed these immune-depressive effects (*p* < 0.001; Figures 7B,D,F,H). Hence, the activation status of distinct cells in intestinal as well as systemic compartments is down-regulated by broad-spectrum antibiotic treatment, but can be restored upon VSL#3 application or FMT.

### Memory/effector T cells in intestinal and systemic compartments of secondary abiotic mice following recolonization with VSL#3 or fecal microbiota transplantation

**Figure 8:**
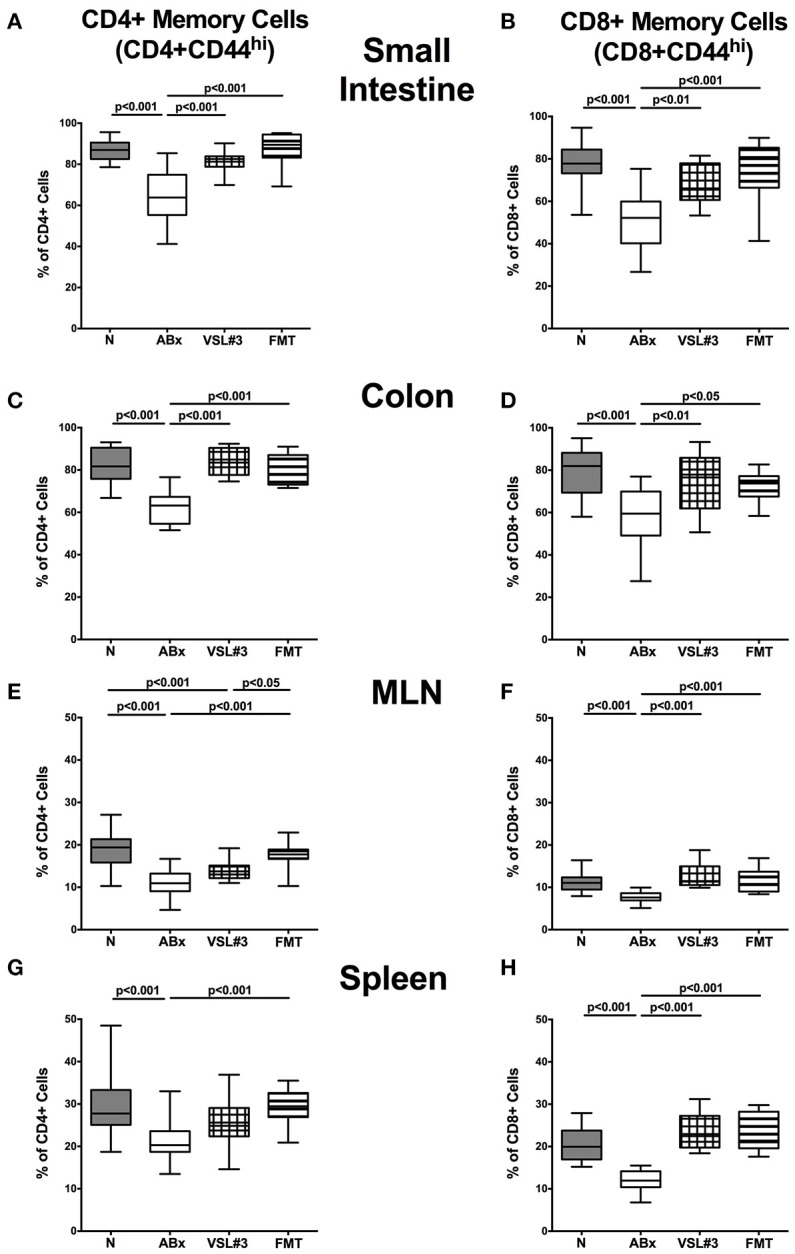
**Memory and effector T cells in intestinal and systemic compartments of secondary abiotic mice following recolonization with VSL#3 or complex murine microbiota**. The proportions of CD4+ memory/effector cells (CD4+CD44^hi^, gated on CD4+ cells, left panel **A,C,E,G**) and CD8+ memory/effector cells (CD8+CD44^hi^, gated on CD8+ cells, right panel **B,D,F,H**) in the small intestine **(A,B)**, colon **(C,D)**, MLN **(E,F)**, and spleen **(G,H)** in naive conventional mice (N), by antibiotic treatment generated secondary abiotic mice (ABx), and mice subjected to VSL#3 recolonization or fecal microbiota transplantation (FMT) were determined on day 28 following peroral reassociation. Box plots represent the 75th and 25th percentiles of the medians (black bar inside the boxes). Total range and significance levels (*p*-values) determined with one-way ANOVA test followed by Tukey post-correction test for multiple comparisons are indicated. Data shown were pooled from two independent experiments (*n* = 10–15/group).

We then investigated the impact of VSL#3 and FMT on the memory/effector CD4+ and CD8+ cells by evaluating high expression of CD44, a surface marker expressed (on both, CD4+ and CD8+ cells) upon previous antigen contact (Sprent and Surh, 2002). ABx mice exhibited a significant reduction in abundances of both, CD4+CD44+ and CD8+CD44+ cells in all examined intestinal and systemic lymphoid organs (*p* < 0.001; Figure 8). In the small and large intestines, VSL#3 recolonization and FMT resulted in a strong up-regulation of CD44 expression on CD4+ as well as CD8+ cells (*p* < 0.05–0.001; Figures 8A–D). The same held true for CD8+ cells in MLN and spleen (*p* < 0.001; Figures 8F,H), whereas FMT alone resulted in higher frequencies of memory CD4+ cells in MLN and spleen as compared to antibiotic treatment (*p* < 0.001; Figures 8E,G). Hence, microbial depletion resulted in reduced abundances of memory/effector T cells in intestinal and systemic lymphoid compartments, that could, however, at least in part be restored by VSL#3 treatment or FMT.

### Pro- and anti-inflammatory cytokine production in intestinal and systemic compartments of secondary abiotic mice following recolonization with VSL#3 or fecal microbiota transplantation

**Figure 9:**
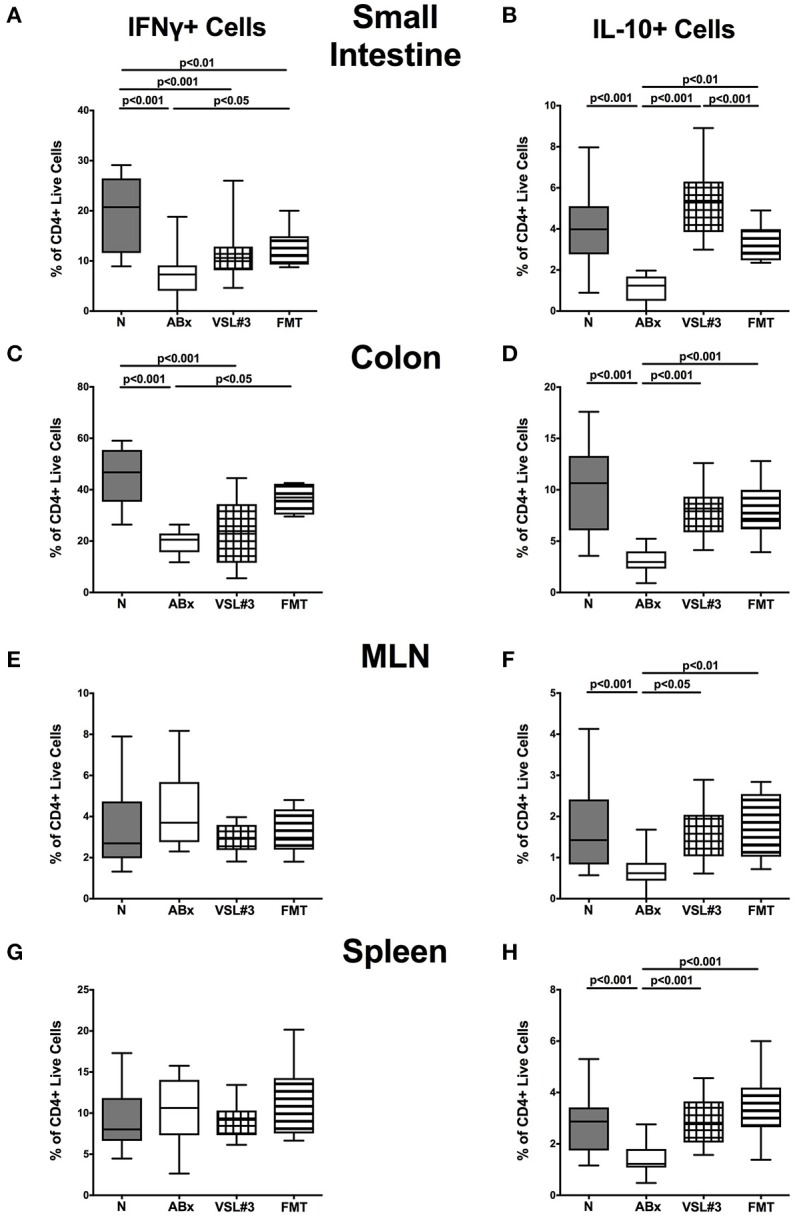
**IFN-γ and IL-10 producing CD4+ cells in intestinal and systemic compartments of secondary abiotic mice following recolonization with VSL#3 or complex murine microbiota**. Lymphocytes were isolated from small intestinal and colonic lamina propria, MLN, and spleen and stimulated with PMA/ionomycin in presence of brefeldin A and subsequently analyzed by flow cytometry. The percentages of IFN-γ (left panel **A,C,E,G**) and IL-10 (right panel **B,D,F,H**) producing CD4+ cells in the small intestine **(A,B)**, colon **(C,D)**, MLN **(E,F)**, and spleen **(G,H)** in naive conventional mice (N), by antibiotic treatment generated secondary abiotic mice (ABx), and mice subjected to VSL#3 recolonization or fecal microbiota transplantation (FMT) were determined on day 28 following peroral reassociation. Box plots represent the 75th and 25th percentiles of the medians (black bar inside the boxes). Total range and significance levels (*p*-values) determined with one-way ANOVA test followed by Tukey post-correction test for multiple comparisons are indicated. Data shown were pooled from two independent experiments (*n* = 10–15/group).

**Figure 10:**
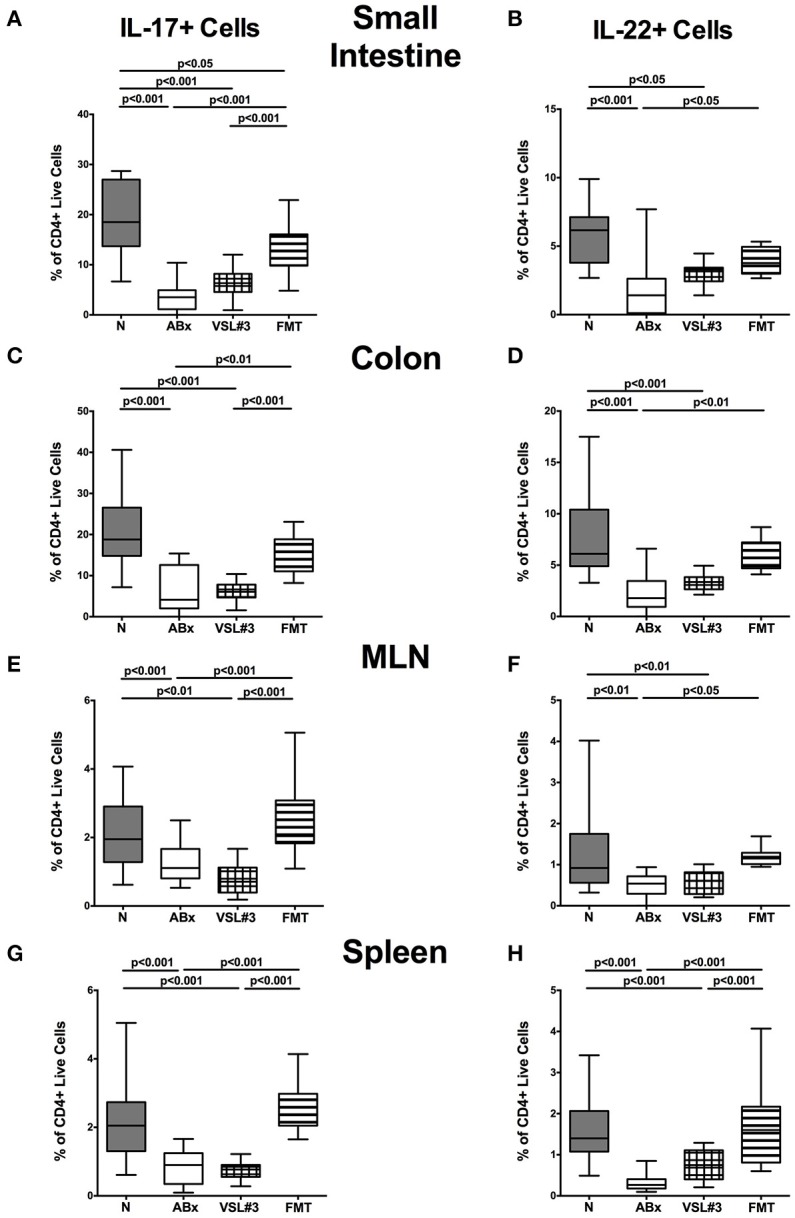
**IL-17 and IL-22 producing CD4+ cells in intestinal and systemic compartments of secondary abiotic mice following recolonization with VSL#3 or complex murine microbiota**. Lymphocytes were isolated from small intestinal and colonic lamina propria, MLN, and spleen and stimulated with PMA/ionomycin in presence of brefeldin A and subsequently analyzed by flow cytometry. The percentages of IL-17 (left panel **A,C,E,G**) and IL-22 (right panel **B,D,F,H**) producing CD4+ cells in the small intestine **(A,B)**, colon **(C,D)**, MLN **(E,F)**, and spleen **(G,H)** in naive conventional mice (N), by antibiotic treatment generated secondary abiotic mice (ABx), and mice subjected to VSL#3 recolonization or fecal microbiota transplantation (FMT) were determined on day 28 following peroral reassociation. Box plots represent the 75th and 25th percentiles of the medians (black bar inside the boxes). Total range and significance levels (*p*-values) determined with one-way ANOVA test followed by Tukey post-correction test for multiple comparisons are indicated. Data shown were pooled from two independent experiments (*n* = 10–15/group).

We further assessed the cytokine producing properties of CD4+ T lymphocytes following VSL#3 administration or FMT in ABx mice. Therefore, we determined the frequencies of IFN-γ and IL-10 (Figure 9) as well as of IL-17 and IL-22 (Figure 10) producing CD4+ cells in small and large intestines, MLN, and spleens. Gating strategies are depicted in Figures S3G–I and representative dot plots shown in Figure S4. Small intestinal IFN-γ producing CD4+ cells were depressed in ABx mice (*p* < 0.01–0.001; Figure 9A) and could not be fully recovered by either bacterial recolonization regimen. Notably, small intestinal CD4+IFN-γ+ cells were more frequently abundant following FMT as compared to ABx mice (*p* < 0.05; Figure 9A). Moreover, IFN-γ producing CD4+ cells were less abundant in large intestines of ABx and VSL#3 treated, but not with fecal microbiota transplanted mice (*p* < 0.05–0.001; Figure 9C), whereas CD4+IFN-γ+ cells did not differ between respective groups in MLN and spleen (n.s.; Figures 9E,G). A strong reduction of CD4+ lymphocytes producing the anti-inflammatory cytokine IL-10 could be determined in all immunological sites following antibiotic therapy (*p* < 0.001; Figures 9B,D,F,H). Notably, reassociation with either VSL#3 or complex murine microbiota could fully restore the frequencies of CD4+IL10+ cells in all compartments (*p* < 0.001; Figures 9B,D,F,H) and thus reestablish the pre-antibiotic (naive) status. Interestingly, intestinal as well as systemic CD4+ cells producing the pro-inflammatory cytokines IL-17 or IL-22 were less abundant upon antibiotic and also subsequent VSL#3 treatment (*p* < 0.05–0.001; Figures 10A–H), but not upon FMT, except for small intestinal CD4+IL17+ cells (*p* < 0.05; Figure 10A).

**Figure 11:**
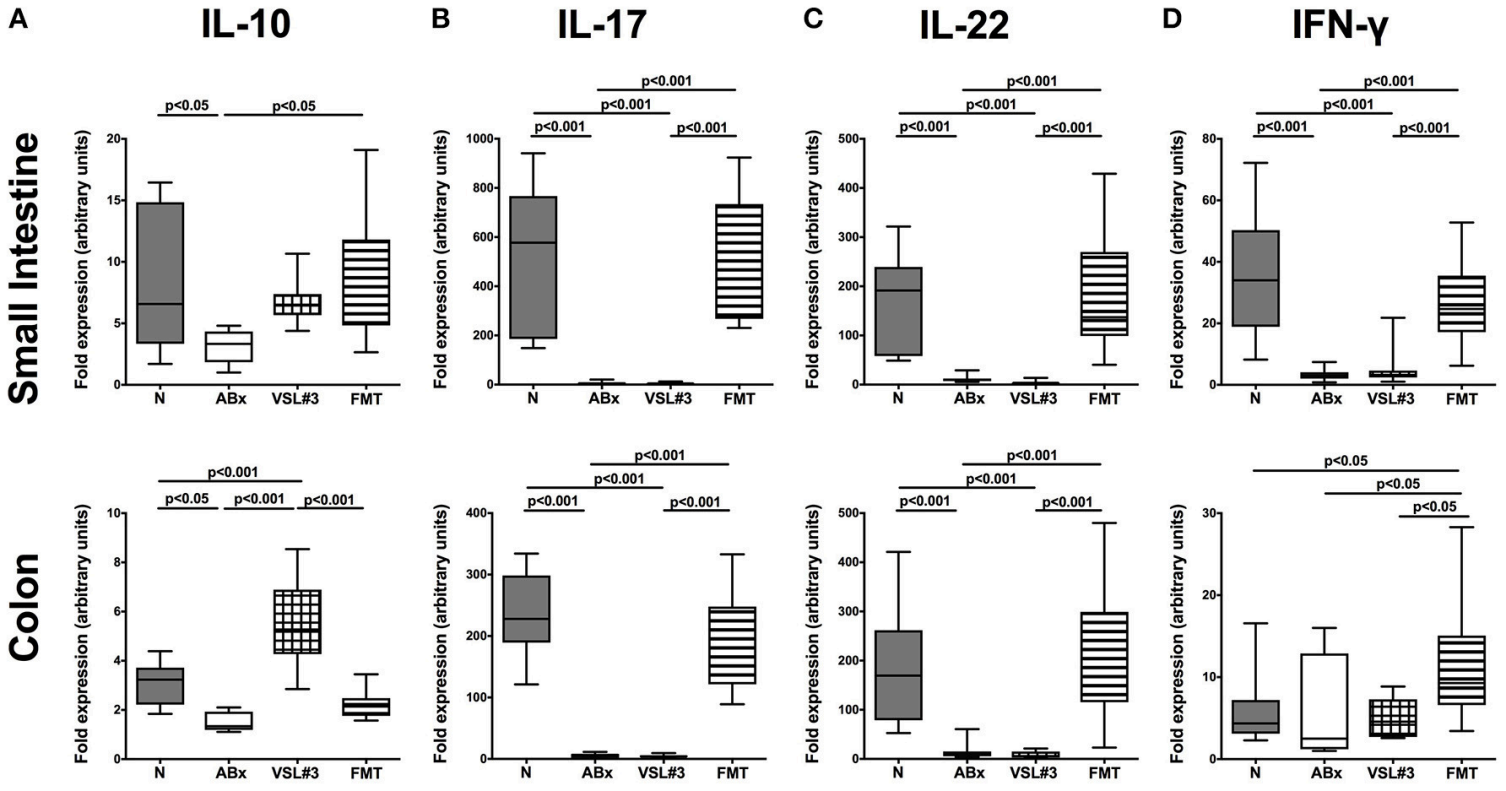
**mRNA analysis of pro- and anti-inflammatory cytokines in small intestinal and colonic tissues**. RT-PCR results of **(A)** IL-10, **(B)** IL-17, **(C)** IL-22, and **(D)** IFN-γ expression in small intestinal (upper panel) and colonic (lower panel) tissues derived from naive conventional mice (N), from by antibiotic treatment generated secondary abiotic mice (ABx), and from mice subjected to VSL#3 recolonization or fecal microbiota transplantation (FMT) on day 28 following peroral reassociation are depicted. Box plots represent the 75th and 25th percentiles of the medians (black bar inside the boxes). Total range and significance levels (*p*-values) determined with one-way ANOVA test followed by Tukey post-correction test for multiple comparisons are indicated. Data shown were pooled from two independent experiments (*n* = 10–15/group).

These findings were further underlined by results obtained from mRNA analysis of respective cytokines measured in ileal and colonic *ex vivo* biopsies (Figure 11). IL-10 as well as IL-17, IL-22, and IFN-γ mRNA were all down-regulated in both small and large intestines of ABx mice (*p* < 0.05–0.001; Figure 11). In the small intestine, FMT (*p* < 0.05), but not VSL#3 treatment could sufficiently up-regulate IL-10 expression back to naive levels, whereas the other way around, recolonization with VSL#3 (*p* < 0.001), but not with complex SPF microbiota reversed antibiotics-induced colonic IL-10 down-regulation (Figure 11A). Furthermore, IL-17 and IL-22 mRNA expression were down-regulated upon antibiotic treatment and also subsequent VSL#3 administration (*p* < 0.001; Figures 11B,C), whereas respective cytokine levels were comparable in mice subjected to FMT and naive controls, which also held true for ileal IFN-γ mRNA expression (Figure 11D). Notably, colonic IFN-γ mRNA levels were highest in mice following FMT (*p* < 0.05), but did not differ between secondary abiotic, VSL#3 treated and naive mice (Figure 11D).

Taken together, peroral probiotic VSL#3 treatment and FMT are both sufficient to induce regulatory, anti-inflammatory mechanisms of the peripheral and central immune system and to restore intestinal as well as systemic immunological collateral damages of broad-spectrum antibiotic treatment.

## Discussion

With increasing robust evidence regarding the indispensability of gut inhabiting bacteria in host physiology and their contributions to a plethora of pathologies, potentially beneficial modulations of intestinal microbiota composition have raised interest in clinical research and application (McCarville et al., 2016). Various environmental factors can lead to alterations of the intestinal microbiota composition, including diet, pathogens, toxins, and drugs (Carding et al., 2015). One of the most prominent factors causing perturbation of this well-balanced and sensitive ecological system is the widespread usage of antibiotics worldwide. Though being very effective in curing infectious diseases and having contributed tremendously to the increased life expectancy, the long-term adverse effects of antimicrobial compounds have been also recognized and explored (Becattini et al., 2016). Yet the underlying mechanisms of the complex crosstalk between microbiota, host, and potential disruptive factors including antibiotic compounds are incompletely understood. Hence, it remains of utmost interest not only to further elucidate them, but also to develop novel therapeutic approaches to alleviate the potential harm exerted by antibiotics. In context of the latter, the study of probiotics, their impact, efficacy, but also limitations is still a challenging and not fully explored field of research.

In the present study we focused on VSL#3, a well-known and clinically approved commercially available probiotic compound, and its impact on the immune system of conventional mice and assessed its efficacy in reversing immunological effects of microbiota depletion as compared to reassociation with a complex murine microbiota. Both complex SPF microbiota and VSL#3 bacteria were able to stably colonize the murine intestinal tract of ABx mice, further supporting the suitability of the microbiota depleted mouse model to explore the complex relationship between the innate and adaptive immune system, antibiotics, and recolonization with defined intestinal bacteria as reviewed by Fiebiger et al. (2016). To assure that the observed immunological responses were merely due to the applied probiotic bacterial species, we performed highly sensitive 16S rRNA based molecular microbiota analyses before and after VSL#3 challenge. In fact, only respective probiotic species and no intestinal bacterial commensals that might have regrown after cessation of antibiotic pretreatment were able to establish in the intestinal tract. Notably, conventionally colonized mice displayed unchanged compositions of their microbiota before and after VSL#3 treatment (our unpublished experimental observations), thus further underlining the physiological colonization resistance that prevents the host from stable pathogenic, but also commensal bacterial colonization (Besselink et al., 2008). Of note, administration of viable microorganisms in an experimental context, especially in studies conducted with immunocompromised individuals, rises safety issues for the prophylactic or therapeutic usage of these compounds. This is supported by former findings of higher mortality in a probiotic intervention group as assessed by a study with critically ill patients suffering from acute pancreatitis (Besselink et al., 2008). However, in our study neither antibiotic treatment nor reassociation with VSL#3 or complex microbiota resulted in any clinical adverse effects in mice such as diarrhea, occurrence of fecal blood or weight loss, nor in microscopic sequelae including apoptosis, indicating that both VSL#3 bacteria and complex microbiota administration are safe and do not cause intestinal inflammation in our applied murine model.

The indispensability of microbial gut stimulation for maintaining epithelial colonic proliferation rates has already been suggested (Reikvam et al., 2011). Our data indicate that VSL#3 treatment of secondary abiotic mice was as effective as reassociation with a complex murine microbiota in stimulating recovery of colonic and ileal epithelial proliferative properties, as indicated by comparably increased Ki67+ cell numbers in intestinal epithelia that were decreased upon quintuple antibiotic therapy. This might be of importance, given that the proliferation of enterocytes is an essential physiological process for tissue repair and maintenance of gut homeostasis, whereas decreased proliferation rates may ultimately result in loss of epithelial integrity (Potten et al., 1997).

To gain an incipient insight into the role of viable VSL#3 bacteria in modulating intestinal immune cells, we quantitatively assessed distinct immune cell populations by applying immunohistochemical analyses of small and large intestinal paraffin sections *in situ*. Interestingly, the mere withdrawal of the antibiotic compounds did not result in restoring small and large intestinal Treg, T and B lymphocytes, implying that the observed immunological responses following VSL#3 or FMT application were attributable to the respective bacterial reassociation.

Overall, our data revealed that the immunomodulatory properties of VSL#3 recolonization were more pronounced in the colon than in the ileum of mice, whereas antibiotic treatment had resulted in decreased small and large intestinal cell numbers of Treg, T and B lymphoctes. While being able to restore the Treg population of the ileum, VSL#3 microbiota could neither induce T nor B lymphocytes in this compartment. In the colon, however, mice harboring VSL#3 bacteria displayed similar numbers of Treg, T and B lymphocytes similar to their naive conventionally colonized or with a complex microbiota recolonized counterparts. These data reemphasize the importance of considering small and large intestine as two distinctive immunological sites with different properties and mechanisms as previously postulated (Mann et al., 2016). Considering that the bacterial loads within the ileum of mice and men range from 10^4^ to 10^8^ CFU per ml luminal content (Quigley and Quera, 2006; Bereswill et al., 2010; Heimesaat et al., 2012), whereas the colonic VSL#3 colonization densities were up to six orders of magnitude higher, might at least in part explain this phenomenon. However, these findings seem to be in contrast to former evidence suggesting the capability of probiotics to modulate ileal immunological responses (Smelt et al., 2013) and prevent from ileitis (Pagnini et al., 2009). Given that *in situ* immunohistochemistry has its spatial limitations and does not provide complete information regarding more complex intestinal immune responses, we performed FACS analysis including not only lymphocytes from the lamina propria of the small and large intestines, but also from the MLN and systemic compartment, namely the spleen, in order to more comprehensively address VSL#3 mediated modulations of immune cell populations.

Remarkably, VSL#3 treatment was more effective in recovering CD4+ and CD8+ lymphocyte numbers in the lamina propria of both mucosal sites as compared to reassociation with complex murine microbiota. Whether this was due to distinct species mediated effects or other underlying mechanisms remains to be further investigated. Moreover, VSL#3 treated mice displayed higher B cell numbers in the small intestinal lamina propria as compared to mice from the other cohorts. Most B cells in the intestinal mucosa are known to be IgA secreting plasma cells (Hill and Artis, 2010). Increased frequencies of small intestinal IgA-expressing B cells have already been demonstrated upon treatment of BALB/c mice with *L. casei* (Galdeano and Perdigon, 2006). Furthermore, a study conducted with intensive care unit patients frequently displaying multiple organ dysfunction as a major cause of mortality (Antonelli et al., 1999) which is pathophysiologically linked to a breakdown of intestinal barrier function and increased translocation of bacteria and bacterial components into the systemic circulation (Hassoun et al., 2001), revealed that serum IgA levels were normalized upon VSL#3 treatment (Alberda et al., 2007). It is therefore tempting to speculate that VSL#3 contributes beneficial effects to host health via IgA mediated mechanisms. Notably, neither reassociation of mice with VSL#3 nor with a complex murine microbiota could reverse the increased splenic CD4+, CD8+, and B220+ cell numbers pointing toward systemic microbiota-independent immunological consequences of long-term broad-spectrum antibiotic therapy. In fact, immunomodulatory properties of distinct antibiotic classes such as quinolones (Dalhoff and Shalit, 2003) and macrolides (Kanoh and Rubin, 2010) have previously been described. In terms of activation status of immune cell populations we could observe that VSL#3 recolonization was as efficient as complex microbiota recolonization, given that VSL#3 application resulted in a complete recovery of Treg, activated DC, and CD8+ memory/effector cells in all included intestinal and systemic immunological sites and of the CD4+ memory/effector cells in the small and large intestinal lamina propria. The latter population, however, could only be fully restored in MLN and spleen upon recolonization with a complex murine microbiota. Similarly, VSL#3 recolonization led to an increase of IL-10 producing CD4+ cell population in all organs, but did not induce the production of pro-inflammatory cytokines such as IL-17, IL-22, and IFN-γ in any of them. In fact, mice harboring VSL#3 bacteria exerted lower intestinal pro-inflammatory cytokine expression levels than their naive or with SPF microbiota recolonized counterparts. To further substantiate these findings, we additionally performed mRNA expression analysis of respective cytokines in ileal and colonic *ex vivo* biopsies and obtained similar results. Hence, our data suggest that VSL#3 dominated microbiota selectively induces activation of memory/effector T cells, activated DC and Treg, and the key anti-inflammatory cytokine IL-10 without driving pro-inflammatory Th1 or Th17 type immune responses. The concept of VSL#3 inducing regulatory immune responses in Th1 or Th17 mediated immune diseases has already been proposed (Di Giacinto et al., 2005) and is, in fact, very attractive for clinical application. Another probiotic mixture consisting of *Lactobacillus acidophilus, L. casei, L. reuteri, Bifidobacterium bifidium*, and *Streptococcus thermophilus*, has also been demonstrated to induce regulatory DC and Treg and, in turn, to suppress experimental immune disorders such as TNBS colitis, experimental atopic dermatitis and rheumatoid arthritis (Kwon et al., 2010). Given the important role of the Th1 and Th17 cell compartments in the protection against bacterial and fungal pathogens (Aujla et al., 2007), the lack of recovery of IL-17, IL-22, and IFN-γ expressing CD4+ cells upon VSL#3 reassociation raises the question, whether mice harboring probiotic bacteria were only more susceptible to pathogenic infections. The fact that probiotics have been shown to exert inflammation ameliorating effects in with antibiotics pre-treated patients suffering from *Clostridium difficile* toxin associated diarrhea, for instance, does not support this hypothesis (Selinger et al., 2013). The mechanisms underlying health-beneficial probiotic bacterial actions are manifold. Firstly, probiotics have been shown to inhibit growth, metabolism and adhesion of enteropathogenic bacteria (Bernet-Camard et al., 1997; Hudault et al., 1997; Gopal et al., 2001). Furthermore, in competition for nutrients and niches they prevent the host from stable pathogenic colonization (Wagner et al., 2009). Moreover, VSL#3 bacteria have been shown to restore epithelial barrier functions and to stimulate intestinal epithelial TNF production under inflammatory conditions (Pagnini et al., 2009). It is thus rather plausible that, while not inducing inflammatory immune responses in healthy mice (as shown here), VSL#3 may still sufficiently induce pro-inflammatory immune responses against invading pathogens.

In summary, in the present study we provide evidence, that beyond the already proposed immunomodulatory effects of VSL#3 on intraepithelial innate immunity (Pagnini et al., 2009), respective probiotic bacterial species modulate innate and adaptive immune cell populations not only at mucosal sites, but also in the peripheral (i.e., MLN) and central (i.e., spleen) immune system. Moreover, when compared to complex SPF microbiota, VSL#3 is capable of equally restoring distinct immune cell populations following microbiota depletion and strongly regulating anti-inflammatory immune responses.

We conclude that the probiotic compound VSL#3 may be regarded as an effective therapeutic tool to restore immune functions following antibiotic therapy. However, future research is needed to elucidate the distinct molecular mechanisms underlying the interactions between host microbiota, its modulations by antibiotic and/or probiotics and immunity.

## Author contributions

IE: Performed experiments, analyzed data, wrote paper. EK and UF: Performed experiments, analyzed data, co-edited paper. CN: Suggested critical parameters in design of experiments, supplied antibodies. PB: Suggested critical parameters in design of experiments, supplied antibodies, co-edited paper. AS: Provided advice in design and performance of experiments. SB: Provided advice in design and performance of experiments, co-edited paper. MH: Designed and performed experiments, analyzed data, co-wrote paper.

## Funding

This work was supported by grants from the German Research Foundation (DFG) to SB (SFB633, TP A7), MH (SFB633, TP B6), UF (SFB633, TP B6), IE and EK (SFB633, Immuco), and from the German Federal Ministery of Education and Research (BMBF) to SB (TP1.1).

### Conflict of interest statement

The authors declare that the research was conducted in the absence of any commercial or financial relationships that could be construed as a potential conflict of interest.

## Supplementary material

Figure S1 **Representative photomicrographs of apoptotic and proliferating epithelial cells as well as of adaptive immune cell subsets in small and large intestines *in situ* following broad-spectrum antibiotic treatment (ABx) and recolonization with VSL#3 or fecal microbiota transplantation (FMT; 100× magnification, scale bar 100 μm)**.

Figure S2 **Adaptive immune cell subsets in small and large intestines *in situ* following quintuple antibiotic therapy**. The average numbers of T lymphocytes (positive for CD3, **A,D**), B lymphocytes (positive for B220, **B,E**), and regulatory T cells (positive for FOXP3, **C,F**) in the small intestinal (upper panel, **A–C**) and colonic (lower panel, **D–F**) tissue of mice following long-term broad-spectrum antibiotic therapy (ABx, white circles) and at d28 after antibiotic withdrawal (ABx %, black circles) in at least six representative high power fields (HPF, 400× magnification) per animal were determined. Medians and significance levels (*p*-values) determined with Mann Whitney *U*-test are indicated. Data shown were pooled from two independent experiments.

Figure S3 **Gating strategies of extracellular stainings on one representative splenic sample after gating for lymphocytes and including only single cells. (A)** Exclusion of dead cells via LIVE/DEAD Fixable Aqua Dead Cell Stain kit (L/D) in the forward scatter (FSC) **(B)** CD4+ and CD8+ cells gated on living cells, **(C)** B cells and activated DC gated on CD4-CD8- cells, **(D)** Treg and **(E)** CD4+ memory/effector cells gated on CD4+ cells, **(F)** CD8+ memory/effector cells gated on CD8+ cells. Gating strategies of intracellular stainings on one representative ileal sample after gating for lymphocytes and including only single cells are illustrated. **(G)** Identification of CD4+ living cells by exclusion of dead cells via LIVE/DEAD Fixable Aqua Dead Cell Stain kit (L/D) **(H)** IFN-γ and IL-10 gating **(I)** IL-17 and IL-22 gating.

Figure S4 **Representative FACS analysis dotplots of intracellular stainings of pro- and anti-inflammatory cytokines in intestinal and systemic compartments in naive conventional mice (N), by antibiotic treatment generated secondary abiotic mice (ABx), and mice subjected to VSL#3 recolonization or fecal microbiota transplantation (FMT)**.

## Abbreviations

ABx: secondary abiotic
BSA: bovine serum albumin
CD: Crohn's disease
CFU: colony forming units
DC: dendritic cells
DTE: dithioerythritol
EDTA: ethylene diamine tetraacetic acid
FCS: fetal calf serum
FMT: fecal microbiota transplantation
HBSS: Hanks balanced salt solution
HPF: high power field
IBD: inflammatory bowel disease
IBS: irritable bowel syndrome
LPL: lamina propria lymphocytes
MLN: mesenteric lymph nodes
PBS: phosphate buffered saline
PMA: phorbol myristate acetate
SPF: special pathogen free
Th: T helper cell
TNBS: trinitrobenzenesulphonic acid
Treg: regulatory T cells
UC: ulcerative colitis.
