# Supplementary material for: The Probiotic Compound VSL#3 Modulates Mucosal, Peripheral, and Systemic Immunity Following Murine Broad-Spectrum Antibiotic Treatment
Source: Front Cell Infect Microbiol. 2017 May 5;7:167. doi: 10.3389/fcimb.2017.00167 (PMC5418240; doi:10.3389/fcimb.2017.00167)
Supplement: Figure S1 — Representative photomicrographs of apoptotic and proliferating epithelial cells as well as of adaptive immune cell subsets in small and large intestines in situ following broad-spectrum antibiotic treatment (ABx) and recolonization with VSL#3 or fecal microbiota transplantation (FMT; 100× magnification, scale bar 100 μm). [file Image1.PDF]

Apoptotic Cells (Casp3+) – Small Intestine

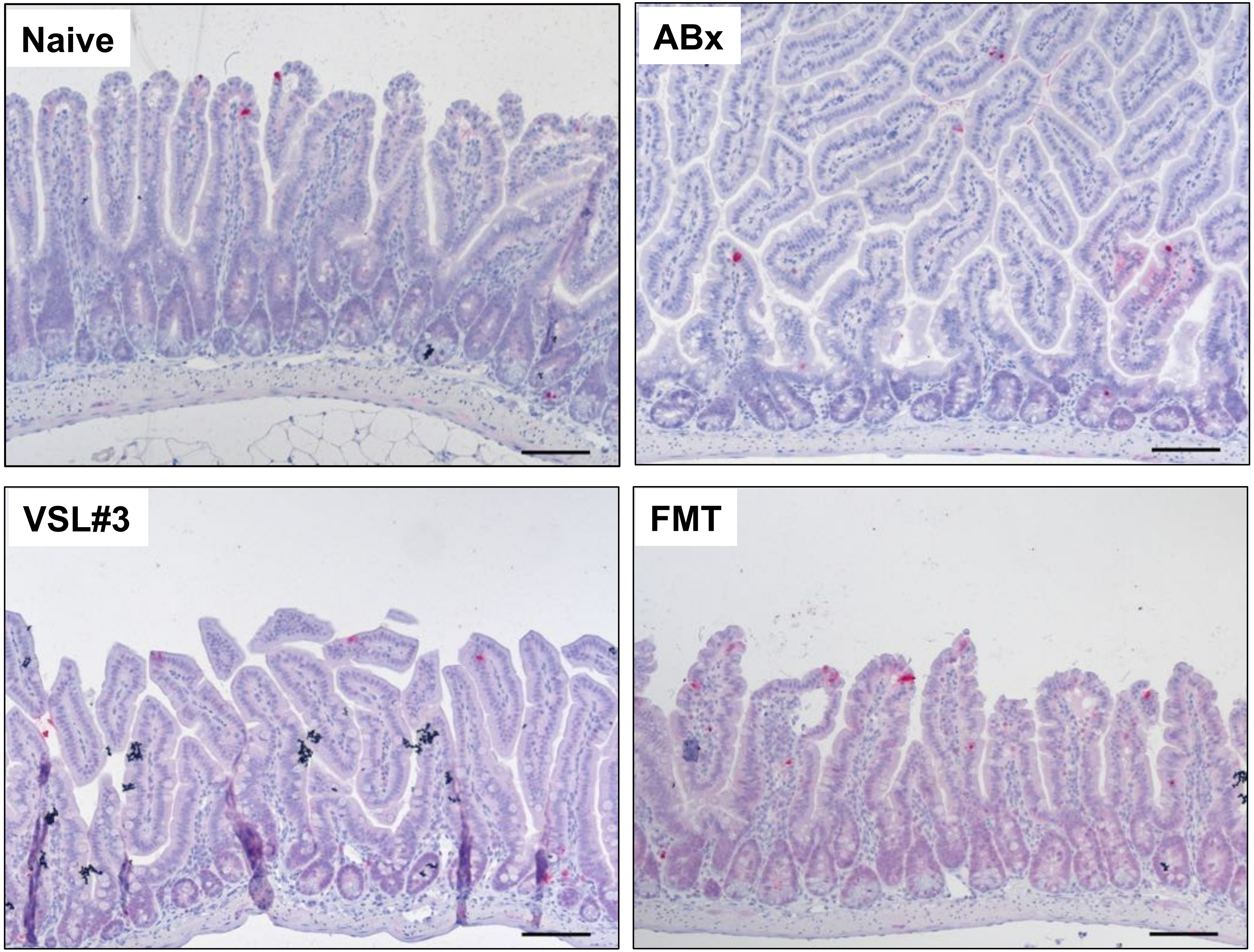

100 x magnification
Scale bar: 100 µm

Apoptotic Cells (Casp3+) – Colon

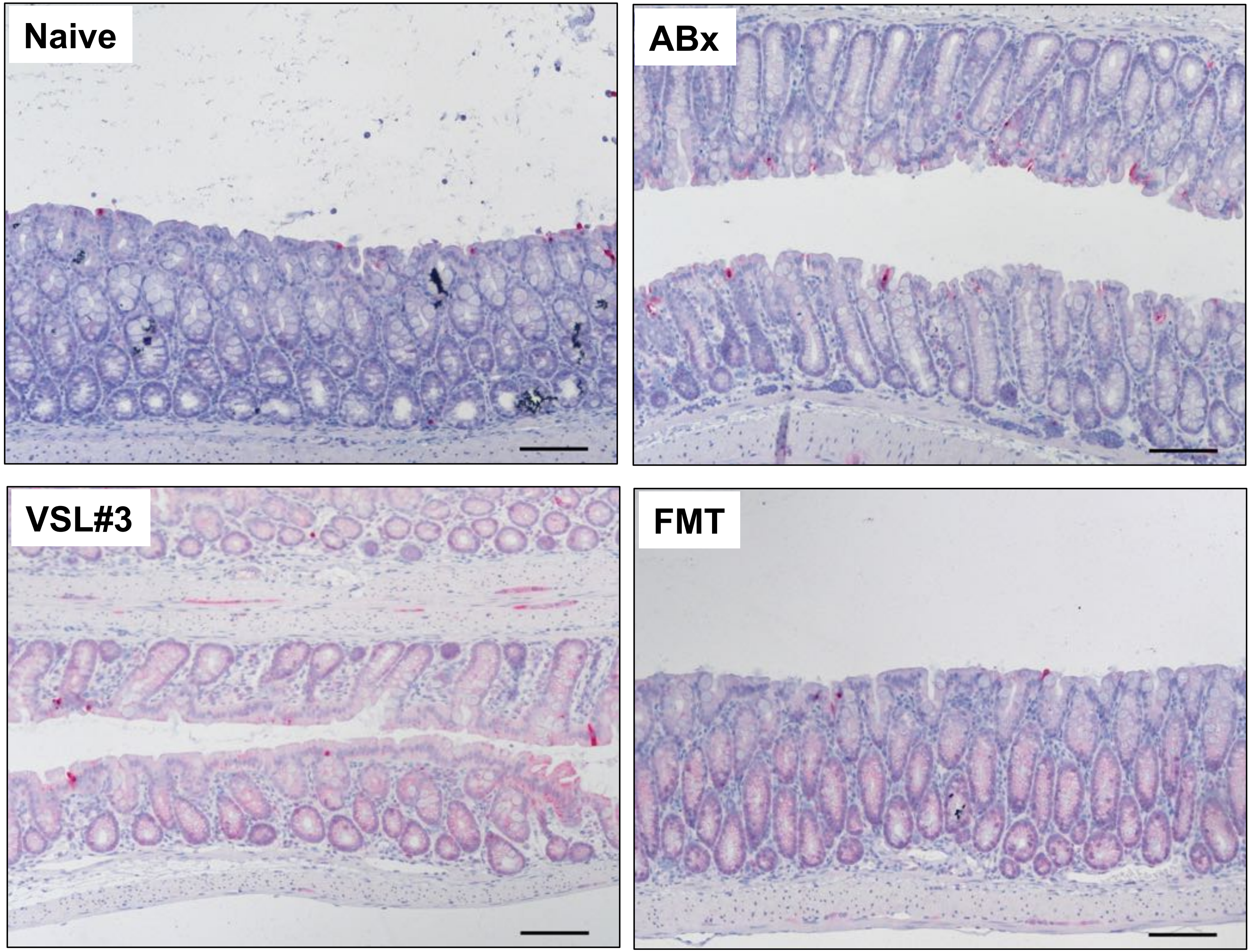

100 x magnification
Scale bar: 100 µm

Proliferating Cells (Ki67+) – Small Intestine

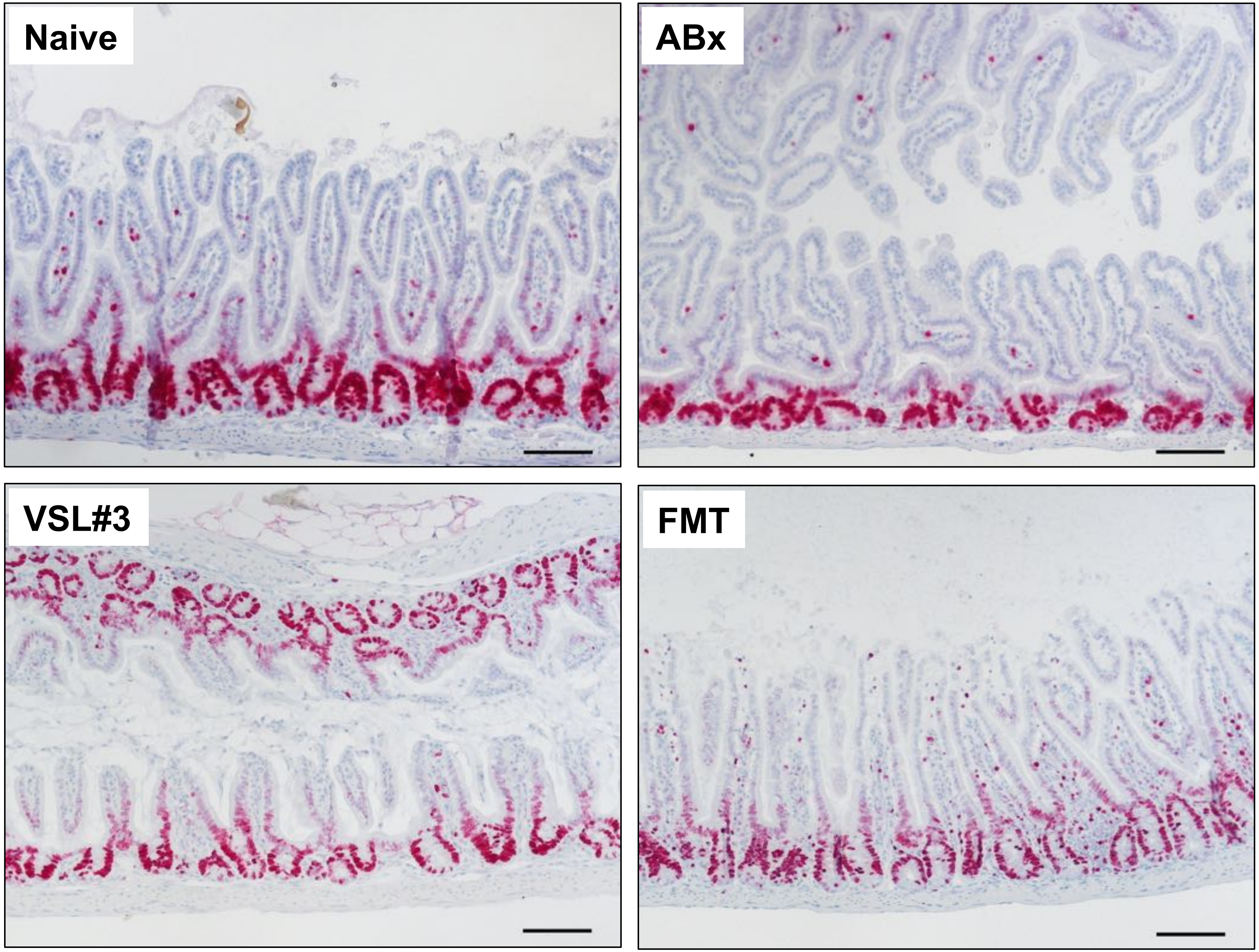

100 x magnification
Scale bar: 100 µm

Proliferating Cells (Ki67+) – Colon

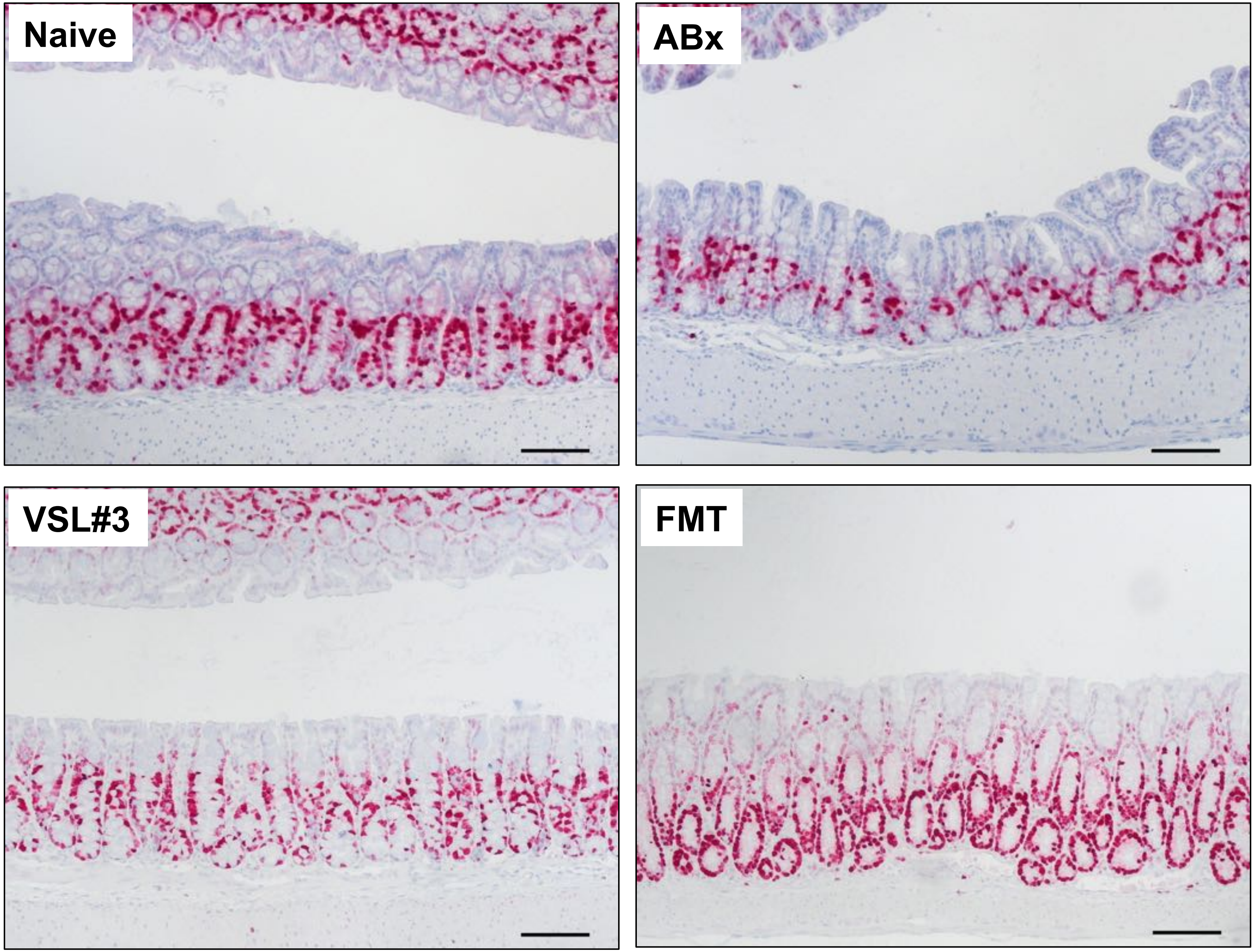

100 x magnification
Scale bar: 100 µm

T Lymphocytes (CD3+) – Small Intestine

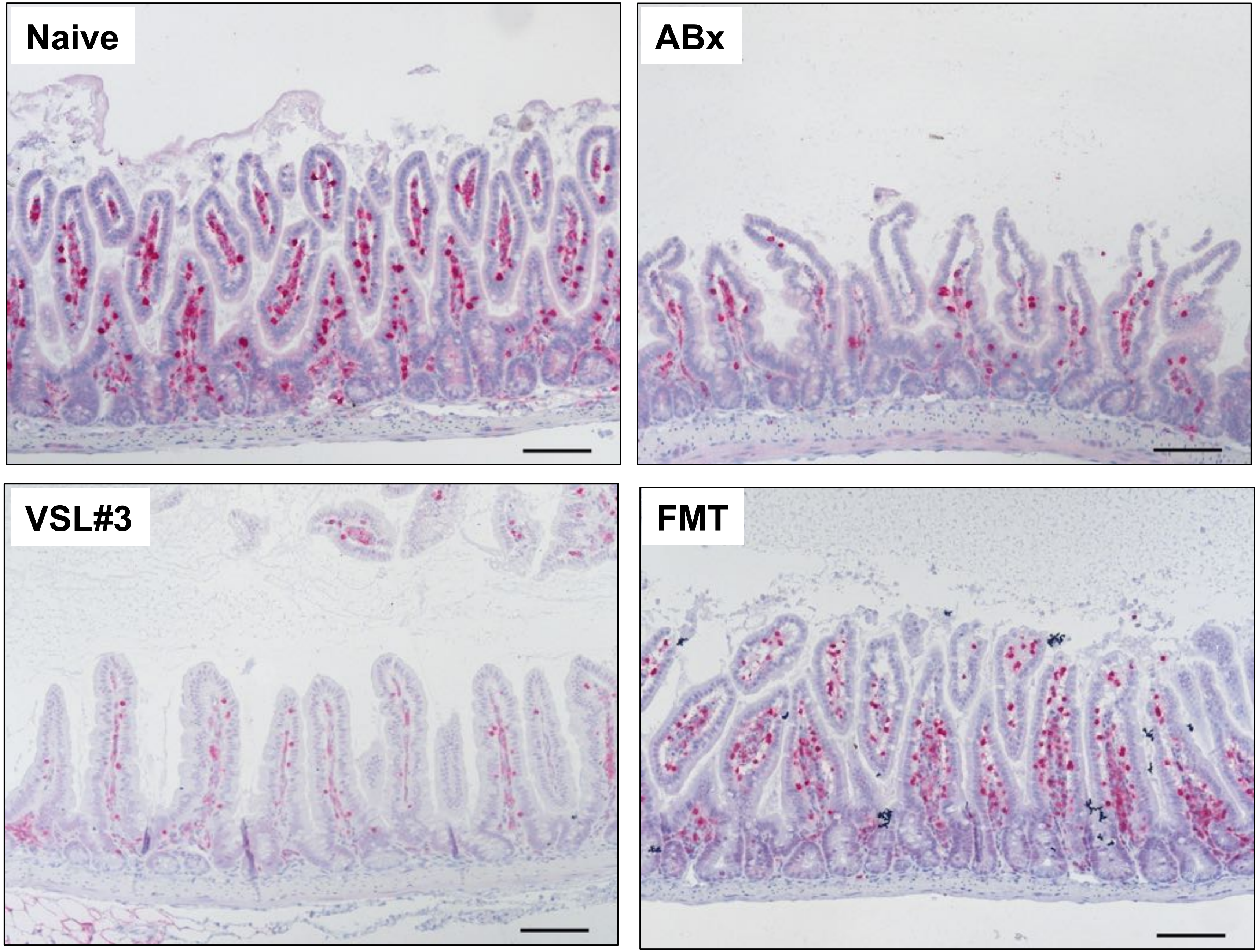

100 x magnification
Scale bar: 100 μm

T Lymphocytes (CD3+) – Colon

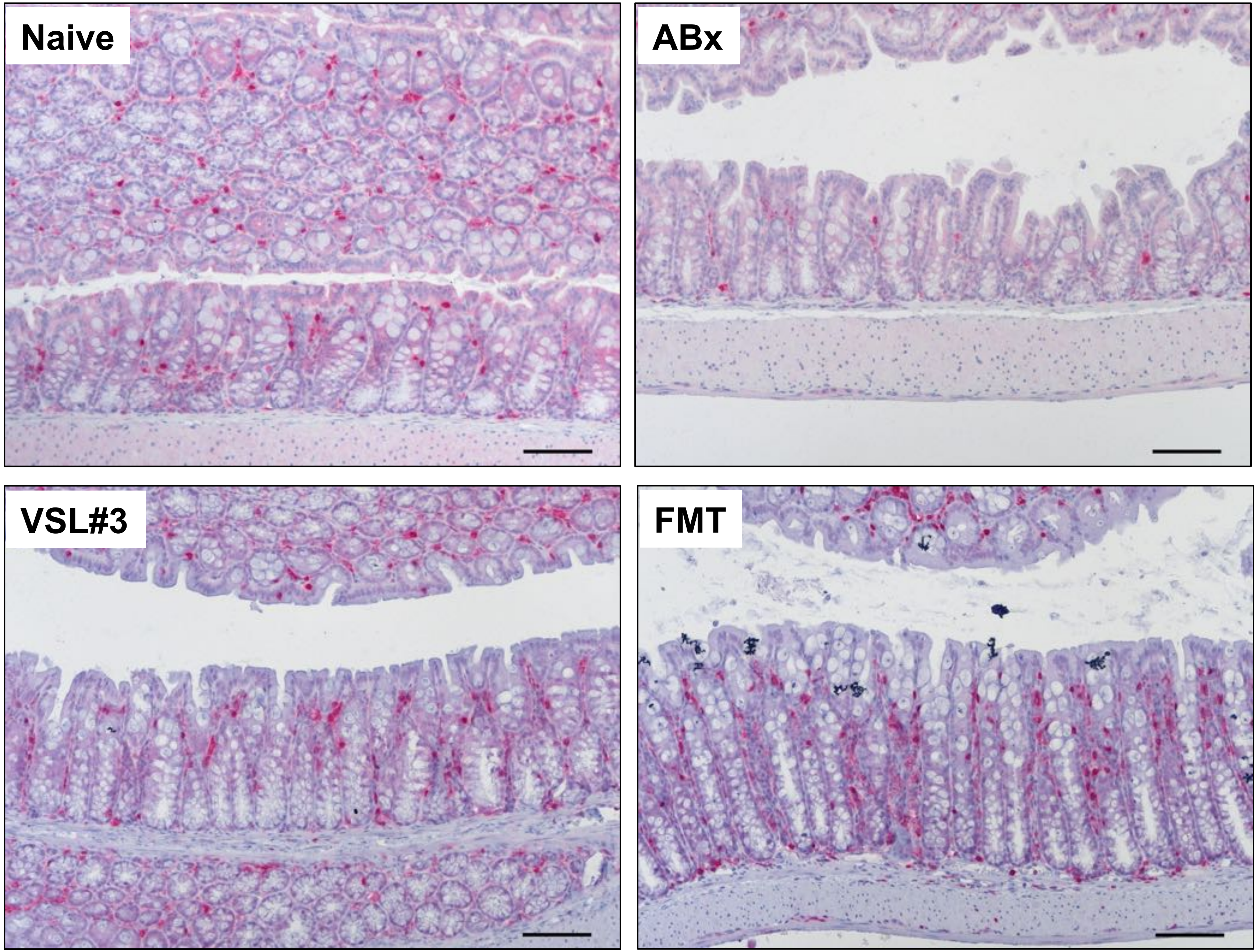

100 x magnification
Scale bar: 100 µm

B Lymphocytes (B220+) – Small Intestine

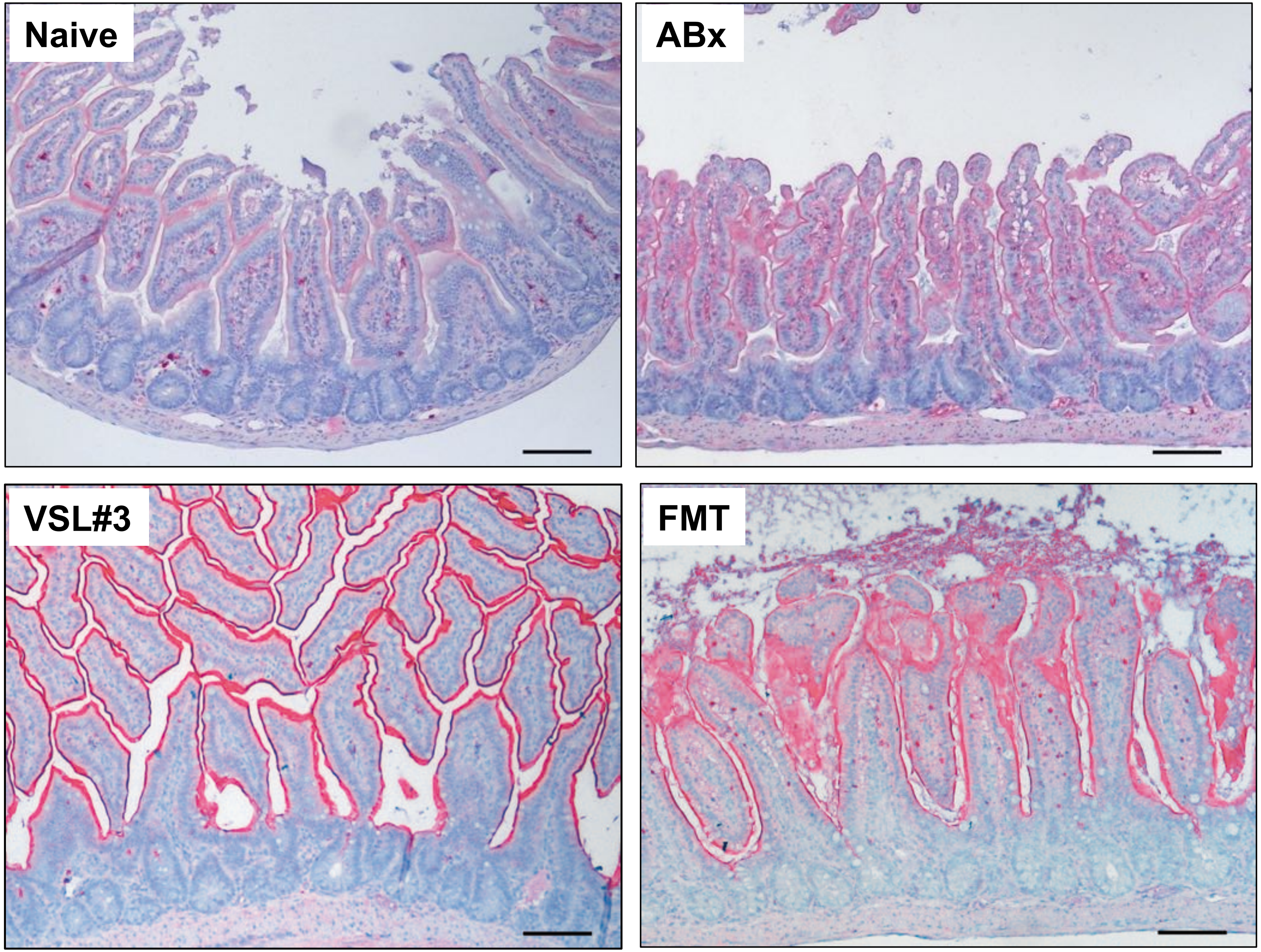

100 x magnification
Scale bar: 100 µm

B Lymphocytes (B220+) – Colon

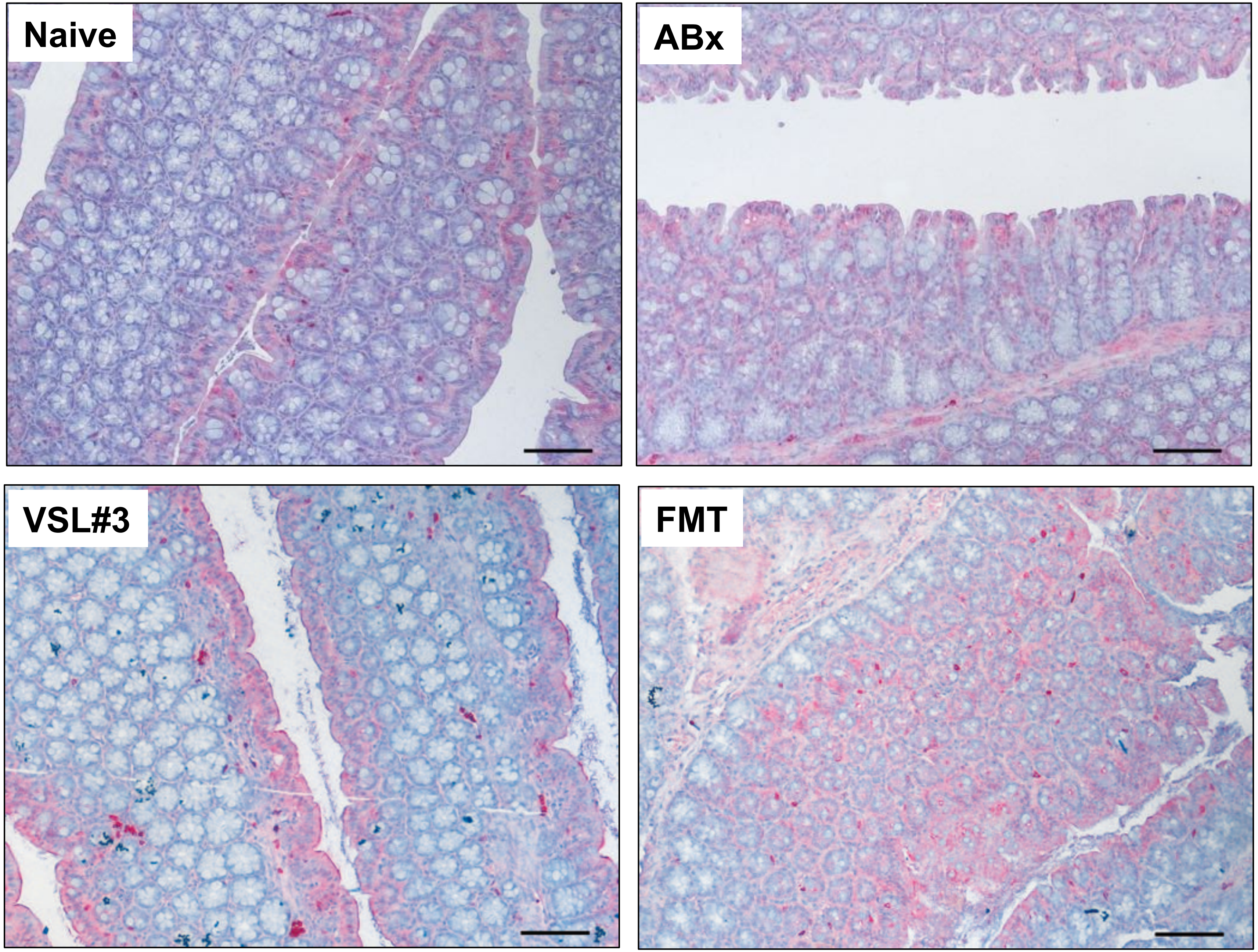

100 x magnification
Scale bar: 100 µm

Regulatory T Cells (Treg, FOXP3+) – Small Intestine

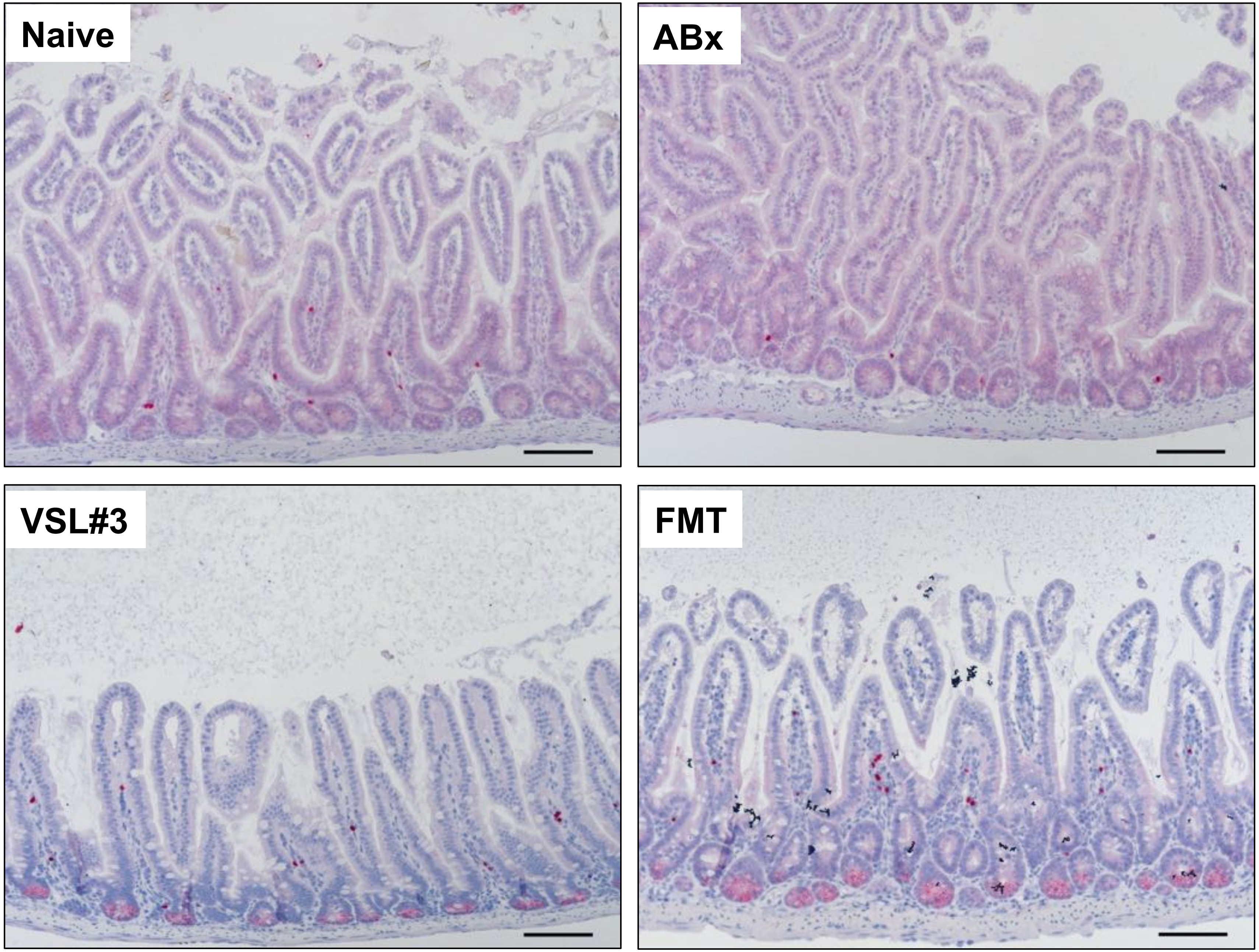

100 x magnification
Scale bar: 100 µm

Regulatory T Cells (Treg, FOXP3+) – Colon

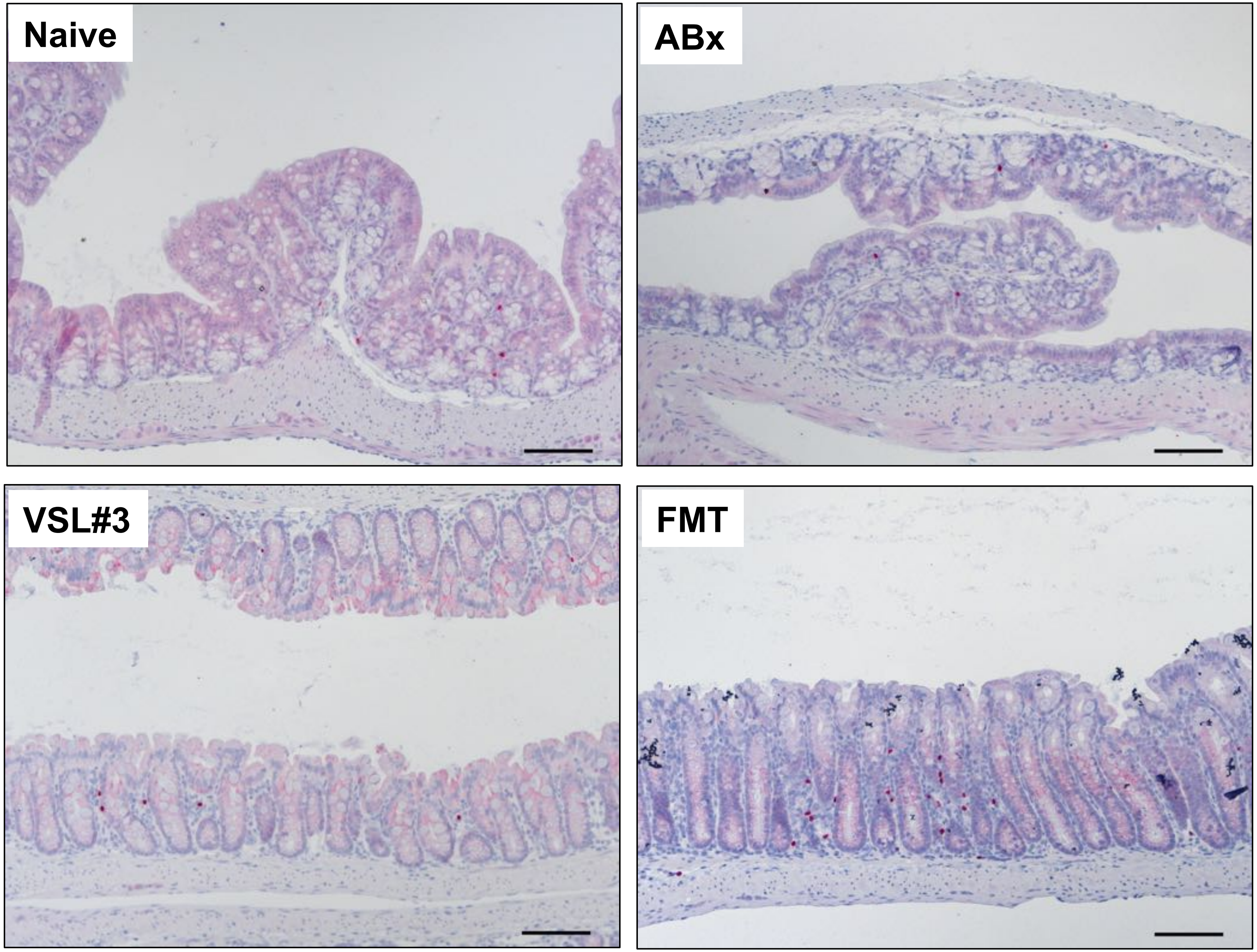

100 x magnification
Scale bar: 100 µm
