# Supplementary figures and images for: The Probiotic Compound VSL#3 Modulates Mucosal, Peripheral, and Systemic Immunity Following Murine Broad-Spectrum Antibiotic Treatment
Source: Front Cell Infect Microbiol. 2017 May 5;7:167. doi: 10.3389/fcimb.2017.00167 (PMC5418240; doi:10.3389/fcimb.2017.00167)

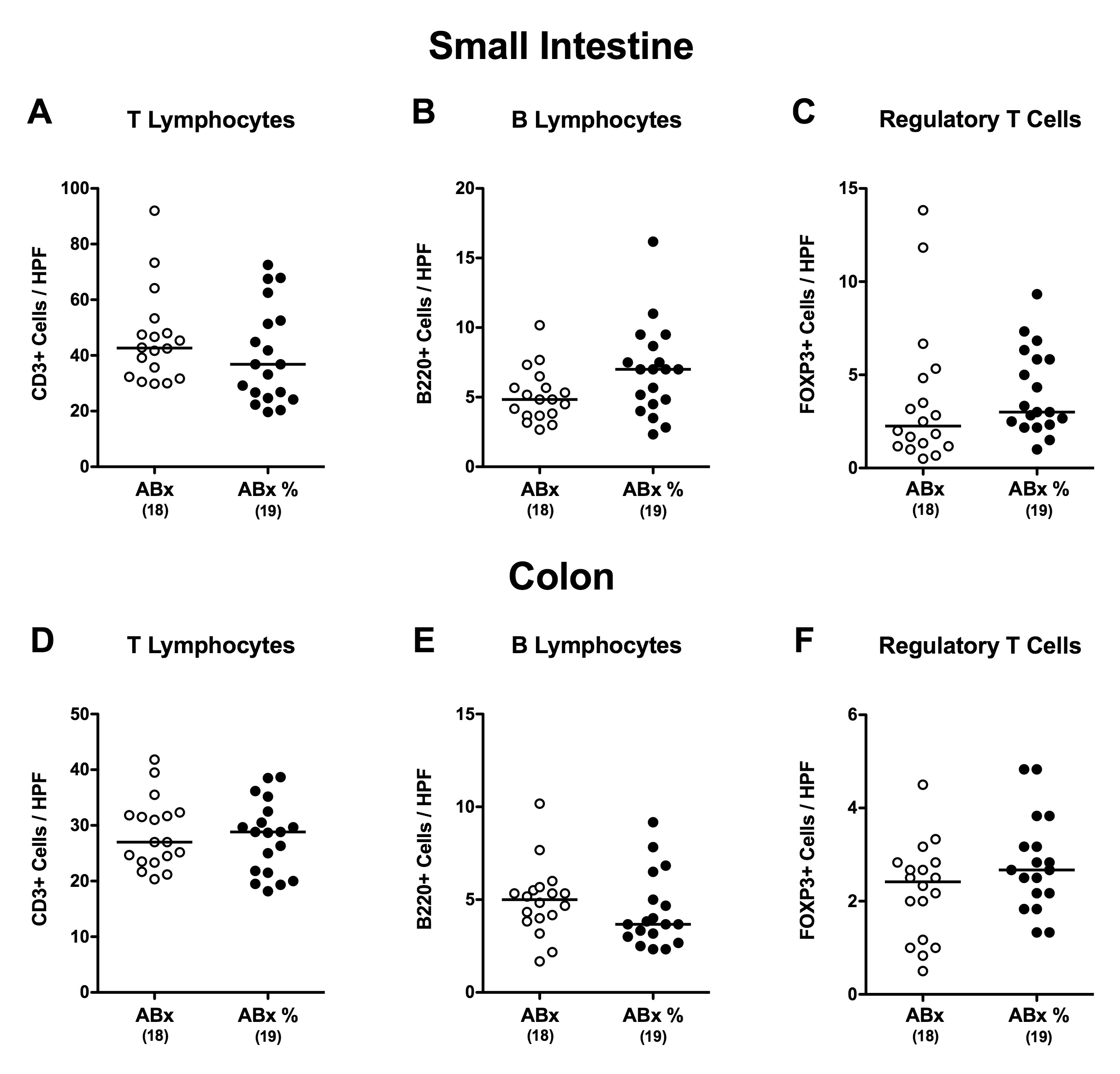

Supplement: Figure S2 — Adaptive immune cell subsets in small and large intestines in situ following quintuple antibiotic therapy. The average numbers of T lymphocytes (positive for CD3, A,D), B lymphocytes (positive for B220, B,E), and regulatory T cells (positive for FOXP3, C,F) in the small intestinal (upper panel, A–C) and colonic (lower panel, D–F) tissue of mice following long-term broad-spectrum antibiotic therapy (ABx, white circles) and at d28 after antibiotic withdrawal (ABx %, black circles) in at least six representative high power fields (HPF, 400× magnification) per animal were determined. Medians and significance levels (p-values) determined with Mann Whitney U-test are indicated. Data shown were pooled from two independent experiments. [file Image2.JPEG]

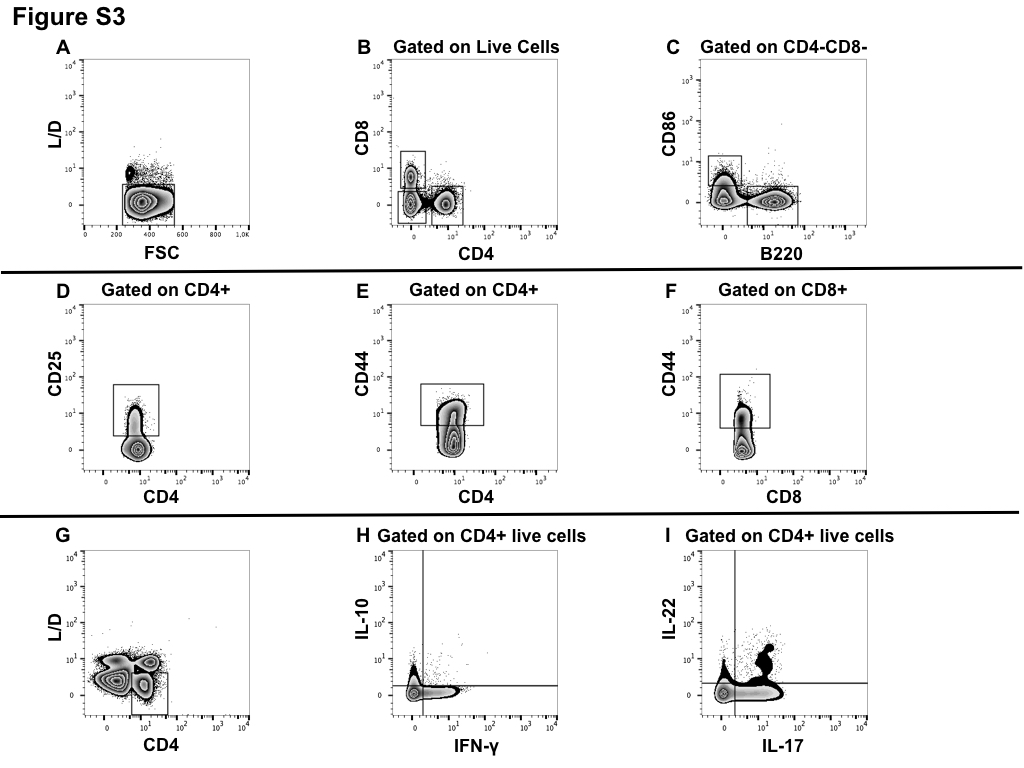

Supplement: Figure S3 — Gating strategies of extracellular stainings on one representative splenic sample after gating for lymphocytes and including only single cells. (A) Exclusion of dead cells via LIVE/DEAD Fixable Aqua Dead Cell Stain kit (L/D) in the forward scatter (FSC) (B) CD4+ and CD8+ cells gated on living cells, (C) B cells and activated DC gated on CD4-CD8- cells, (D) Treg and (E) CD4+ memory/effector cells gated on CD4+ cells, (F) CD8+ memory/effector cells gated on CD8+ cells. Gating strategies of intracellular stainings on one representative ileal sample after gating for lymphocytes and including only single cells are illustrated. (G) Identification of CD4+ living cells by exclusion of dead cells via LIVE/DEAD Fixable Aqua Dead Cell Stain kit (L/D) (H) IFN-γ and IL-10 gating (I) IL-17 and IL-22 gating. [file Image3.JPEG]

**Figure S4**

**Small intestine**

**N**

**ABx**

**VSL#3**

**FMT**

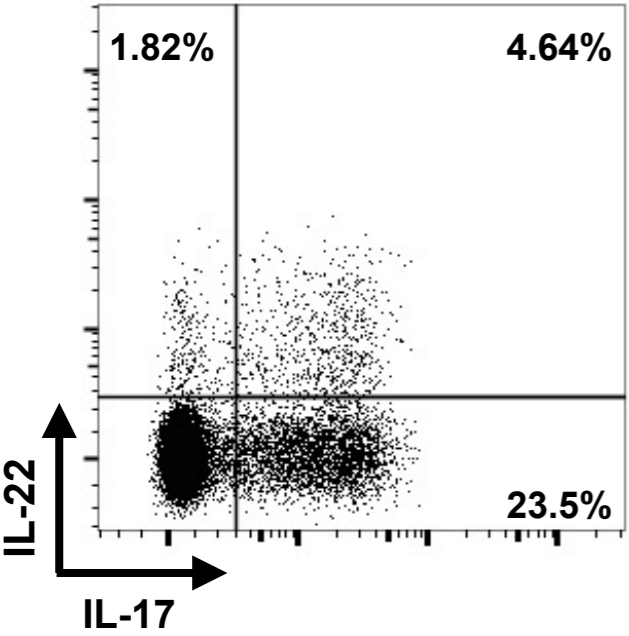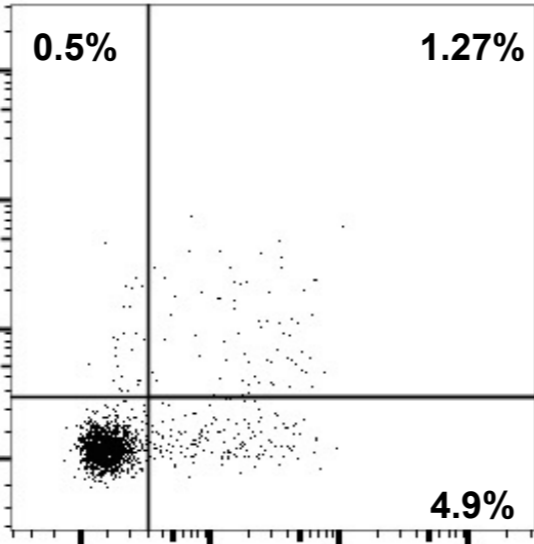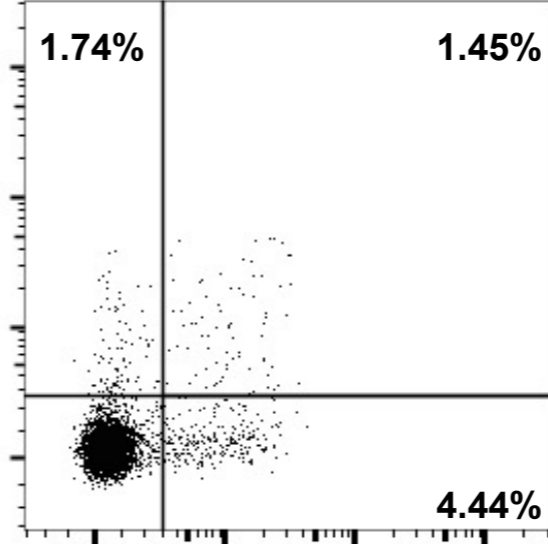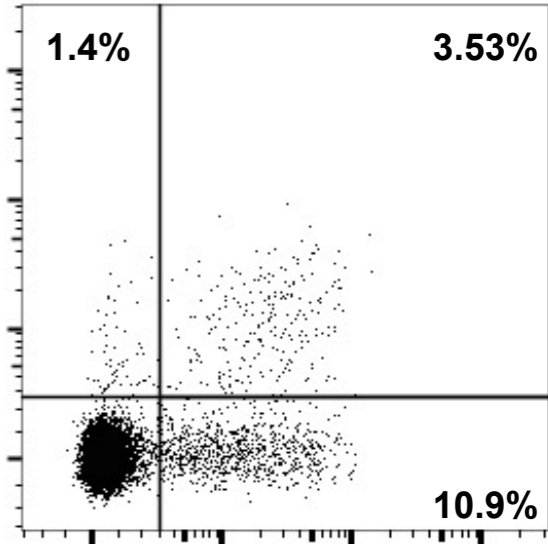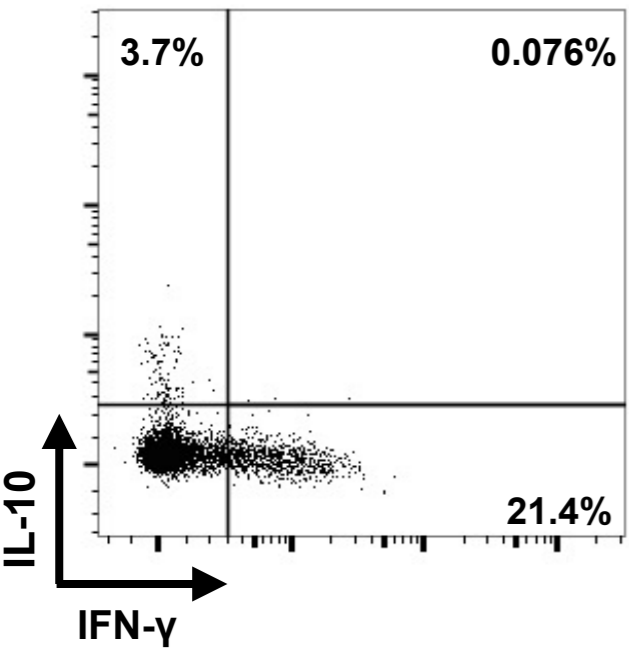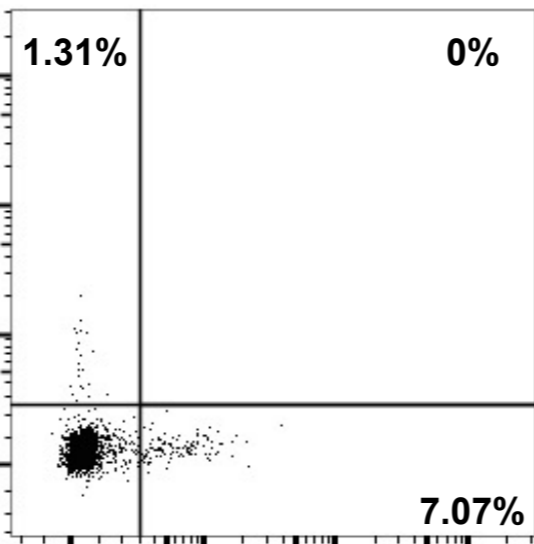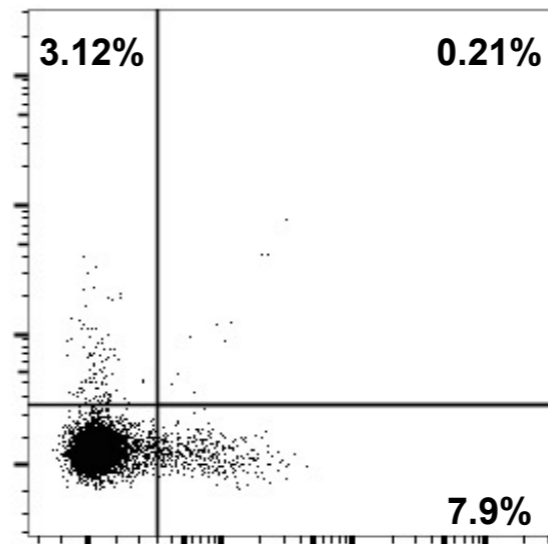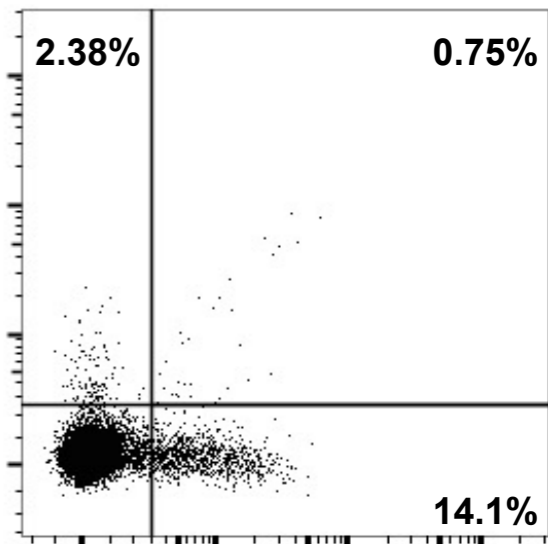

# Colon

**N**

**ABx**

**VSL#3**

**FMT**

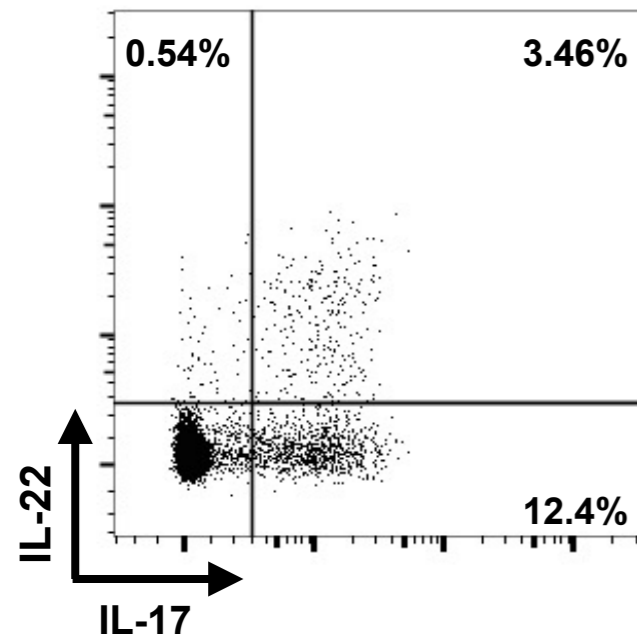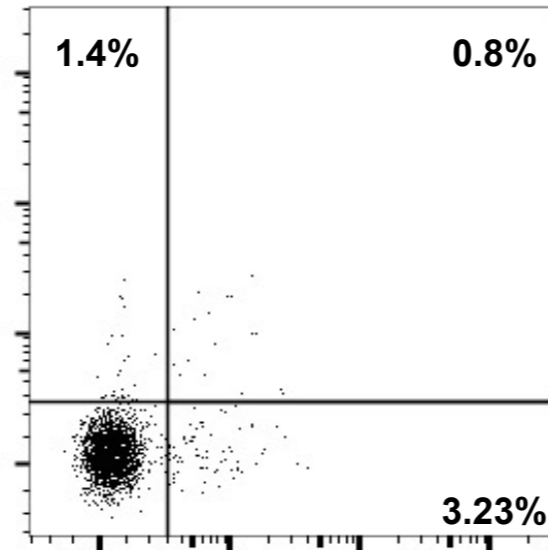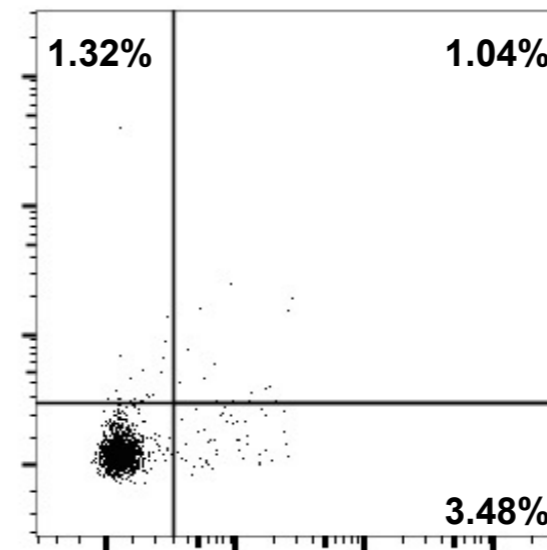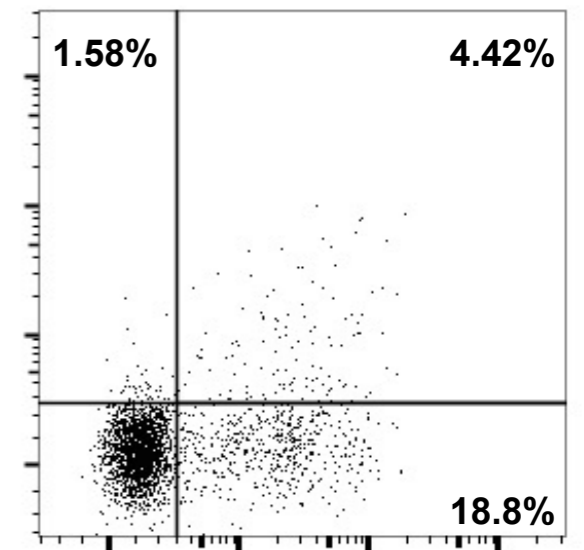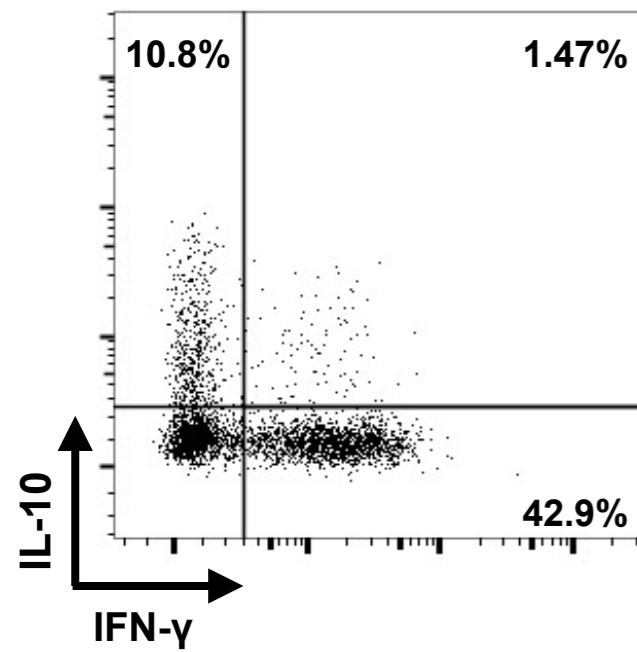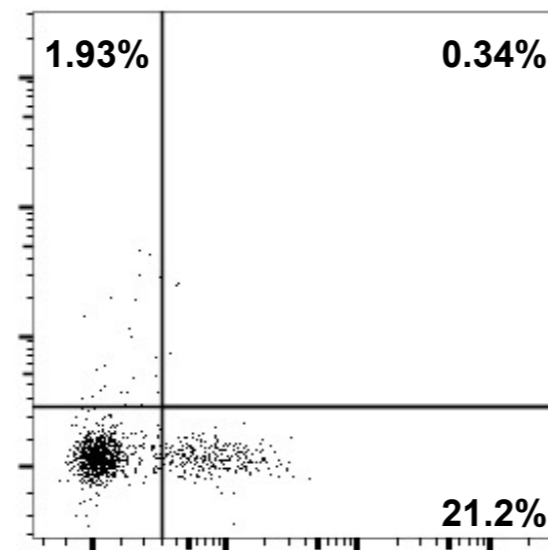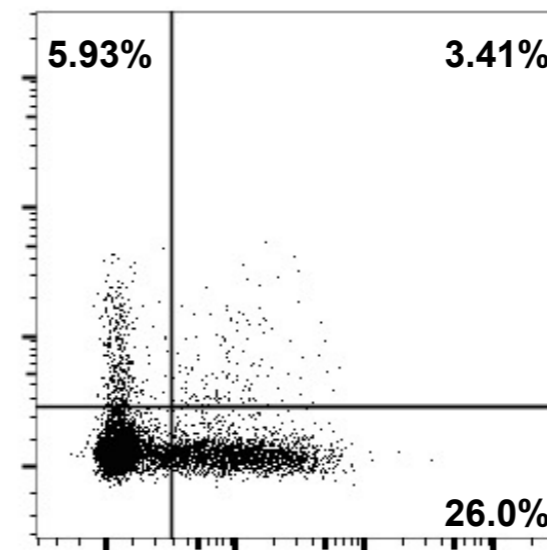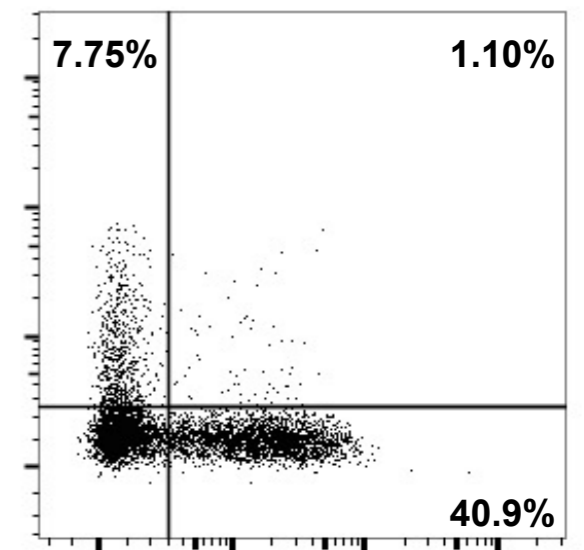

# MLN

**N**

**ABx**

**VSL#3**

**FMT**

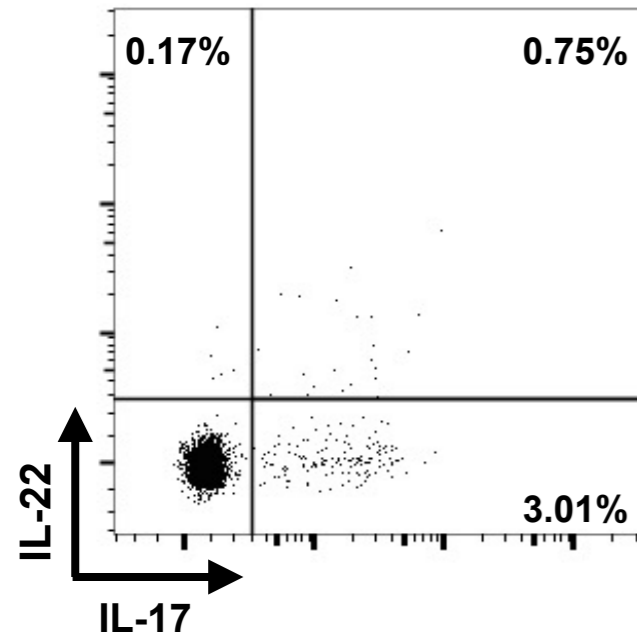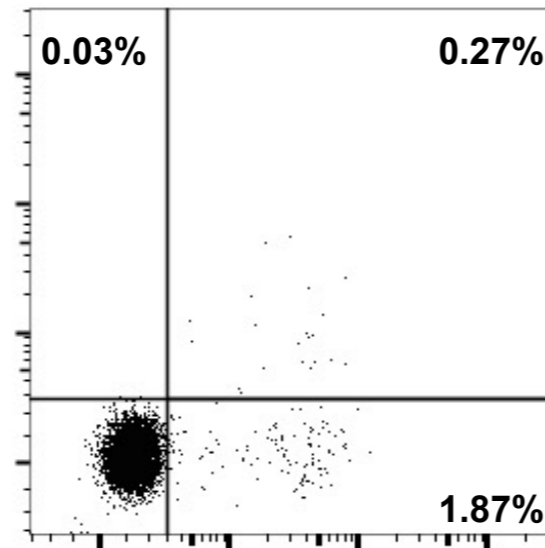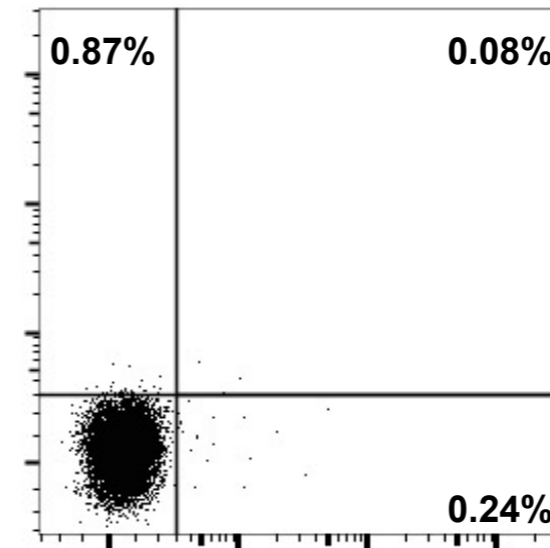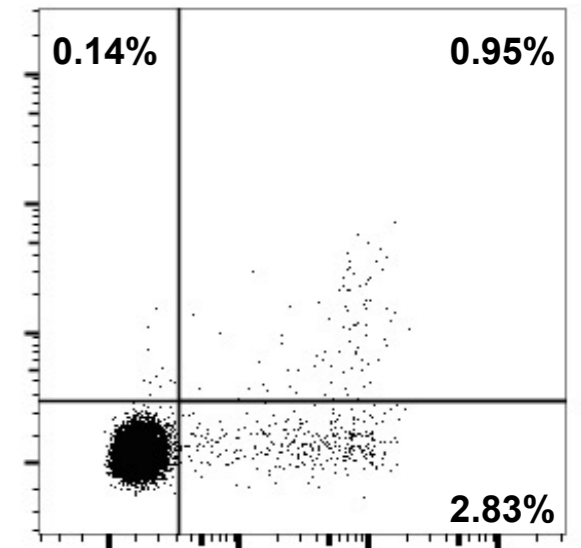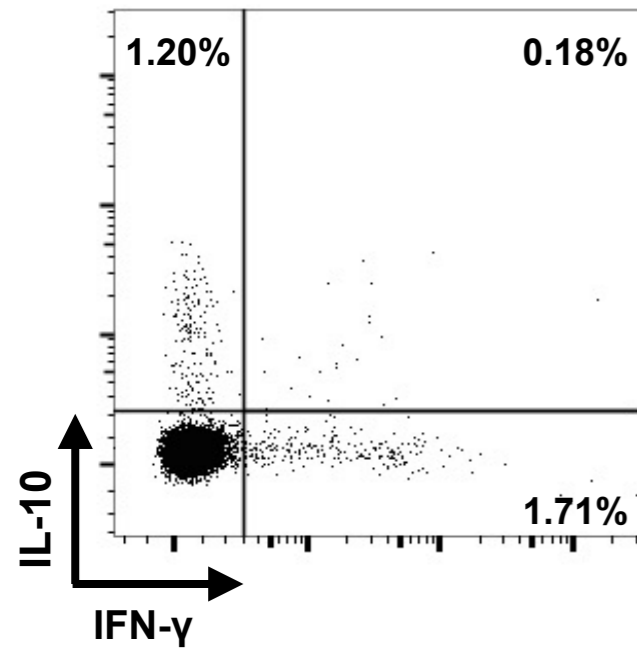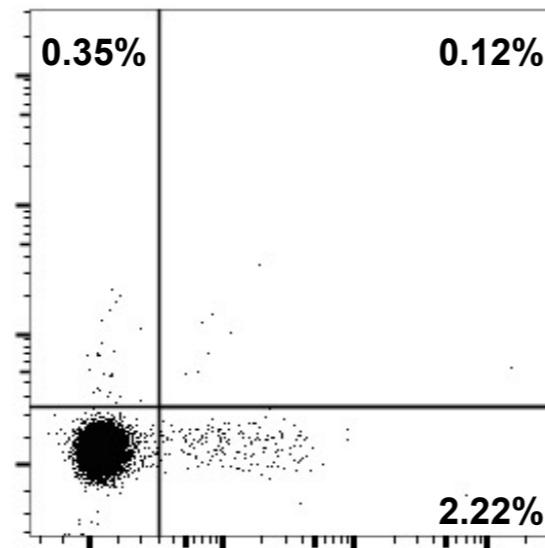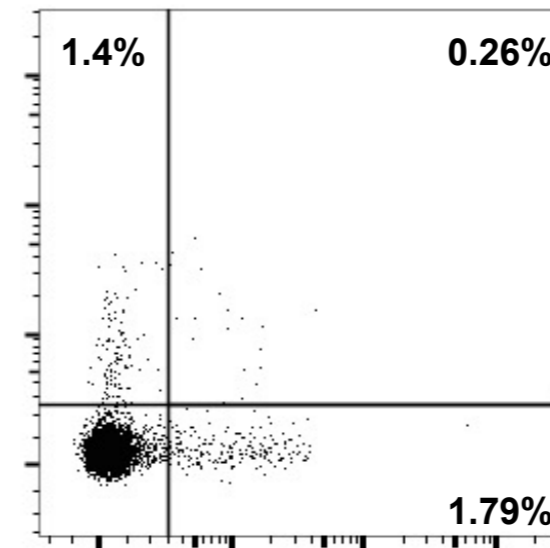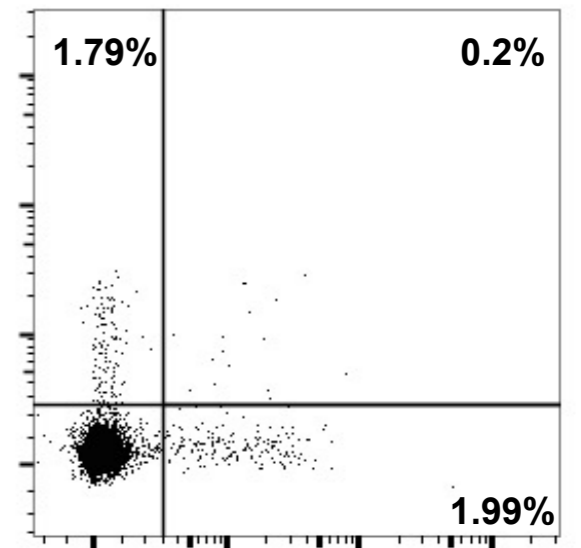

# Spleen

**N**

**ABx**

**VSL#3**

**FMT**

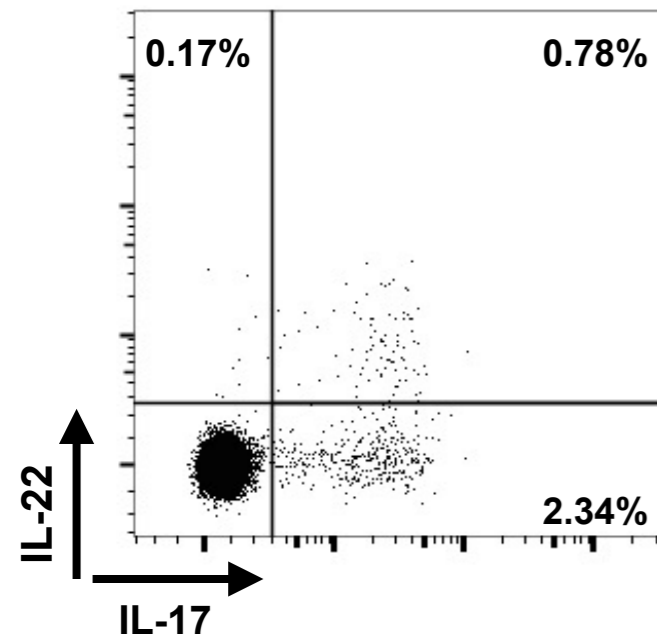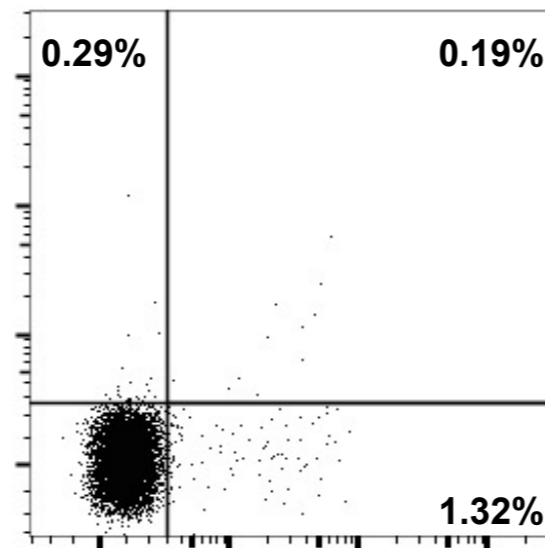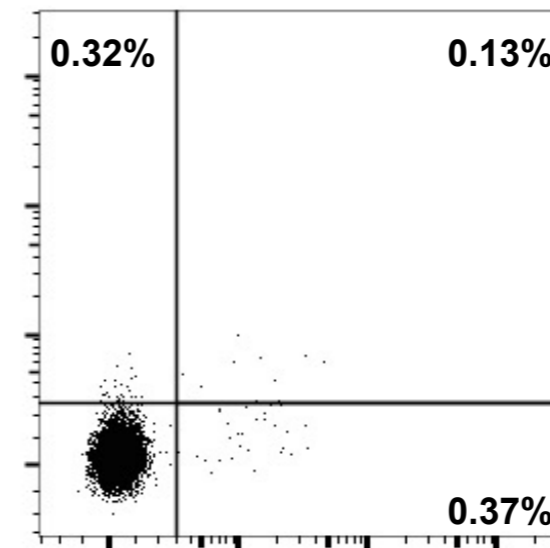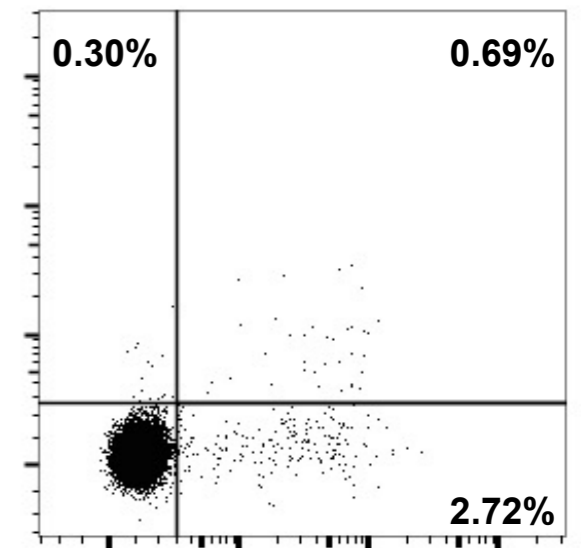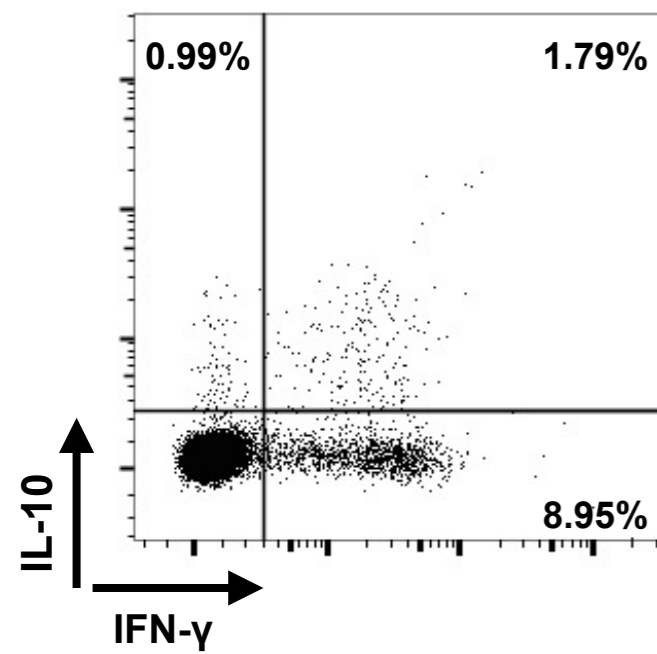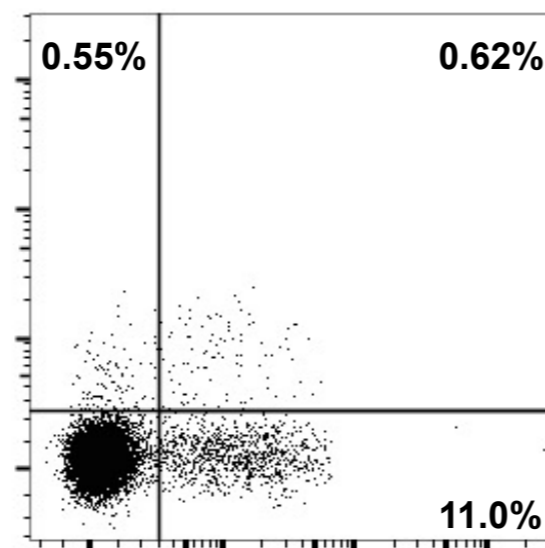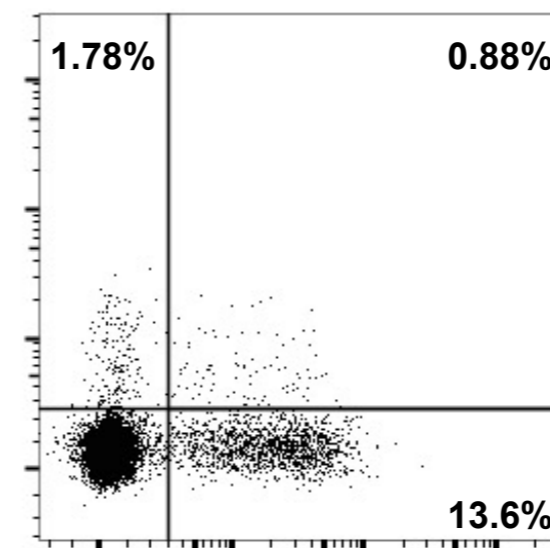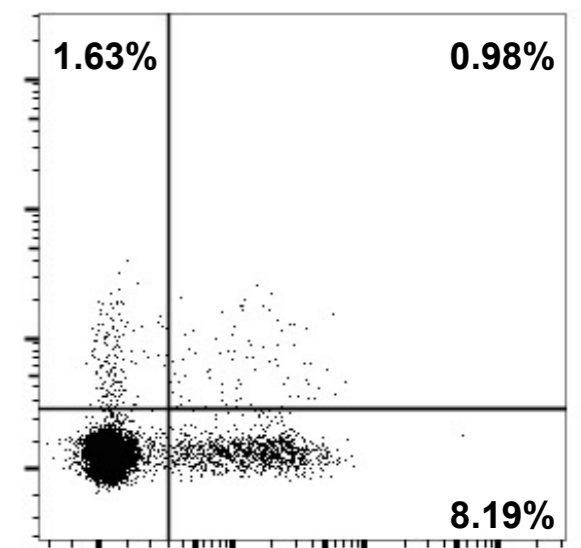

Supplement: Figure S4 — Representative FACS analysis dotplots of intracellular stainings of pro- and anti-inflammatory cytokines in intestinal and systemic compartments in naive conventional mice (N), by antibiotic treatment generated secondary abiotic mice (ABx), and mice subjected to VSL#3 recolonization or fecal microbiota transplantation (FMT). [file Image4.PDF]
